# Microplastic Ingestion Induces Size-Specific Effects in Japanese Quail

**DOI:** 10.1021/acs.est.2c03878

**Published:** 2022-10-27

**Authors:** Laura Monclús, Eliana McCann Smith, Tomasz Maciej Ciesielski, Martin Wagner, Veerle L.B. Jaspers

**Affiliations:** Department of Biology, Norwegian University of Science and Technology (NTNU), Høgskoleringen 5, Trondheim 7491, Norway

**Keywords:** birds, microplastics, in vivo experiment, cytokines, oxidative stress, sex hormones, hepatotoxicity

## Abstract

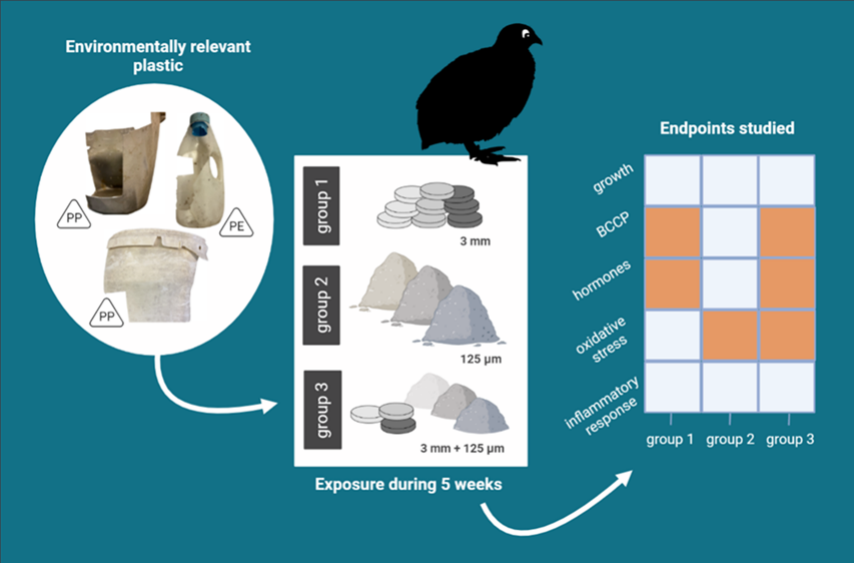

Plastic pollution can pose a threat to birds. Yet, little is known about the sublethal effects of ingested microplastics (MP), and the effects of MP < 1 mm in birds remain unknown. This study therefore aimed at evaluating the toxicity of environmentally relevant polypropylene and polyethylene particles collected in the Norwegian coast in growing Japanese quail (*Coturnix japonica*). Birds were orally exposed to 600 mg MP over 5 weeks, covering small (<125 μm) and large (3 mm) MP, both separately and in a mixture. We evaluated multiple sublethal endpoints in quail, including oxidative stress, cytokine levels, blood-biochemical parameters, and reproductive hormones in blood, as well as body mass. Exposure to small MP significantly induced the activities of the antioxidant enzymes catalase, glutathione-S-transferase, and glutathione peroxidase. Exposure to large MP increased the levels of aspartate aminotransferase (liver parameter) and decreased 17β-estradiol levels in females. Body mass was not directly affected by MP ingestion; however, quail exposed to small MP and a mixture of large and small MP had a different growth rate compared to control quail. Our study used similar levels of MP as ingested by wild birds and demonstrated size-dependent effects of MP that can result in sublethal effects in avifauna.

## Abstract 2

Environmentally relevant microplastics cause oxidative stress, early signs of hepatoxicity, and decreased estradiol concentrations in laboratory exposed quail.

## 1 Introduction

Plastic waste has been accumulating globally over the years and now affects nearly every ecosystem, including marine and freshwater ecosystems, remote regions,^1,2^ and, while less studied, terrestrial environments.^3^ Microplastics (MP, largest diameter <5 mm) arise from the fragmentation of large plastic items driven by natural weathering processes such as biodegradation or UV photodegradation^4^ or are manufactured as primary products from industrial processes.^5^ MP are ubiquitous and persistent in the environment^6−8^ and can pose a risk to many organisms.^9^

The ability of biota to ingest MP has been widely documented;^9^ however, the potential health effects are still not well understood. This is especially true for birds which are one of the taxa most affected by macroplastic and MP ingestion.^10^ Yet, birds are understudied in comparison to fishes and invertebrates.^11−13^ Ingestion of plastic items causes physical harm in birds, including digestive blockages and internal wounds which can affect feeding and energy storage.^14−16^ Most studies have focused on the ingestion of plastic >1 mm in size,^17−21^ likely because of the ease of their identification and long-term accumulation in the gastrointestinal tract.^20^ Moreover, data on MP ingestion in birds mostly come from observational studies that use opportunistic samples from postmortem analysis^22,23^ as well as analysis of feces^2^ and regurgitated pellets.^24,25^ There have been some invasive studies that utilize capture and euthanasia of wild birds,^26^ yet, this method cannot be largely applied because of ethical reasons and the great number of bird species globally threatened.^27^

Since the first experiment with domestic chickens (*Gallus domesticus*) in the 1980s,^28^ only two studies have investigated the effects of MP exposure in birds.^29,30^ While these studies used controlled experiments with Japanese quail (*Coturnix japonica*), another study exposed the streaked shearwater (*Calonectris leucomelas*) to MP in the field and studied the accumulation of plastic chemicals.^31^ Roman et al.^29^ exposed adult quail to 5 and 10 large MP pellets (>3 mm size) and reported minor growth effects in the offspring, delayed sexual maturation in females, and increased frequency of epididymal cysts in males. More recently, de Souza et al.^30^ demonstrated biochemical alterations and oxidative stress in adult quail exposed to 99 and 198 MP of >3 mm size. In the wild, one of the few studies evaluating MP effects in free-living birds (flesh-footed shearwaters *Ardenna carneipes*)^32^ reported alterations in blood-biochemical parameters (BCCPs), although potential field interferences (e.g., malnutrition) were pointed out afterward.^33^ In black vultures (*Coragyps atratus*), plastic ingestion caused oxidative stress, REDOX unbalance, and cholinesterase effects.^34^

These initial studies point toward toxicological effects of MP on avifauna. However, the research on the MP effects on birds is in its infancy, and many questions remain unresolved. One of them is whether small MP have an effect on birds as is known in other vertebrates, such as fishes and mice where particles <125 μm cause oxidative stress^35−37^ and inflammatory reactions.^38,39^ There is growing evidence that some bird species, including raptors,^40^ penguins,^2^ and seabirds,^41^ are exposed to small MP (<1 mm size). Yet, it is unknown if this exposure can result in toxic health effects that could propagate to the individual and population level. Therefore, the matter of MP size can be critically important to assess the risk of MP for birds and direct mitigation and conservation measures.

**Figure 1:**
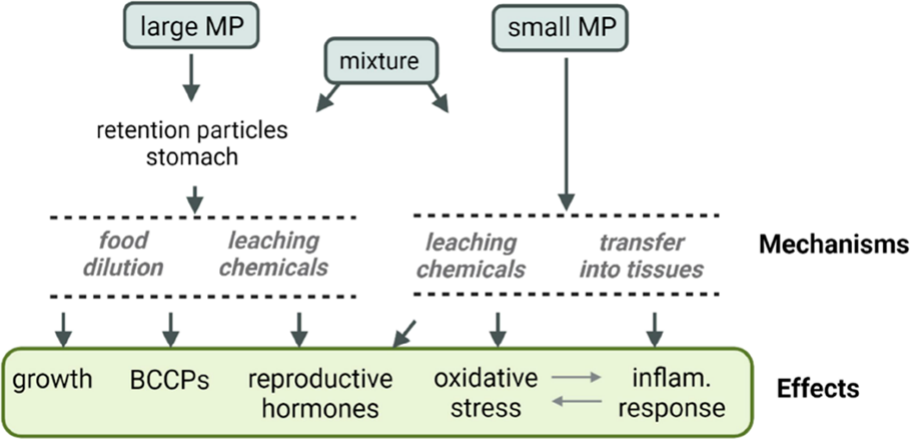
Overview of the hypothesized mechanistic pathways and effects of ingesting large (3 mm) and small (<125 μm) MP. The hypothesized effects associated with the mechanisms were investigated in this study (green highlights). Note: BCCPs = blood-biochemical parameters.

The goal of the present study was to evaluate and differentiate the effects of small and large MP using Japanese quail (hereafter quail) as a bird model. We exposed quail to environmentally relevant doses of polypropylene (PP) and polyethylene (PE) particles produced from plastic collected from the Norwegian coast and investigated the effects of exposure to both large (3 mm) and small (<125 μm) MP in the context of tentative mechanistic pathways (Figure 1).

We hypothesized the following: First, large MP are retained in the stomach and reduce nutrient assimilation (food dilution effect),^28^ which would lead to physiological alterations (e.g., growth, BCCPs, reproductive hormones). Second, small MP cause greater effects on a cellular level (e.g., oxidative stress, inflammatory responses) because of their ability to translocate into tissues.^42^ Third, the mixture of both large and small MP is expected to cause both effects.

## 2 Materials and Methods

This study was approved by the Norwegian Food Safety Authority (Mattilsynet Norway, FOTS ID 24926) and was conducted in the animal facilities at the Department of Biology at NTNU, Norway, according to the appropriate regulations and supervised by accredited personnel (FELASA C).

### 2.1 Plastic Collection and Preparation

Several plastic items were collected from islets in the Mausund archipelago in Norway (63°52’N, 8°39’E) in March 2020. In the lab, the collected items were gently washed with ultrapure water and further analyzed for polymer type. Using a confocal Raman spectrometer (alpha300 R, WITec), the polymer was identified by comparing spectra to the ST-Japan polymer spectra database (L60002). Three items were selected because of their unambiguous polymer identification, high abundance in marine environments,^43^ and large volume available for performing the experiment. These included a white-transparent PE bottle, a gray PP bucket, and a white PP bucket. From these items, two particle size classes were prepared: 3 mm particles (large MP) and <125 μm (small MP). First, the three items were cut into smaller pieces with scissors. Then, pieces were further cut into 3 mm circular disks using a leather hole-puncher. The other half of the pieces were cryo-milled and sieved to obtain particles <125 μm. The size distribution was analyzed using a Coulter counter (Beckman Coulter Multisizer 4e) resulting in an average of 38.52 μm (ranging from 18.78 to 65.26 μm). When preparing and handling the plastic, measures to reduce contamination were implemented (e.g., laboratory cotton coat, rinsing equipment with water and soap between processing different plastic types). More details of the plastic preparation are in Supporting Information (Section 1, Figures S1–S10).

### 2.2 Bird Model and Husbandry

The Japanese quail was selected as a model species for the present study as a well-established model for toxicity studies in avifauna.^44^ Quail have also been used as a terrestrial small-size bird model to clarify the effects of MP in these birds.^29,30^ The quail allow for the study of multiple endpoints in a short period of time (e.g., reproductive and growth parameters, physiology). They are well adapted to captivity and resilient to manipulations^44,45^ and have a fast growth rate and an early onset of maturity (ca. 6 weeks old^45^). We followed established guidelines for the incubation of the eggs and maintenance of the chicks.^45,46^ Throughout the experiment, water and food (starter food for poultry no. 12316, Felleskjøpet, Norway) were provided *ad libitum* (details in Supporting Information Section 2).

### 2.3 Study Design and Exposure to Plastic

**Figure 2:**
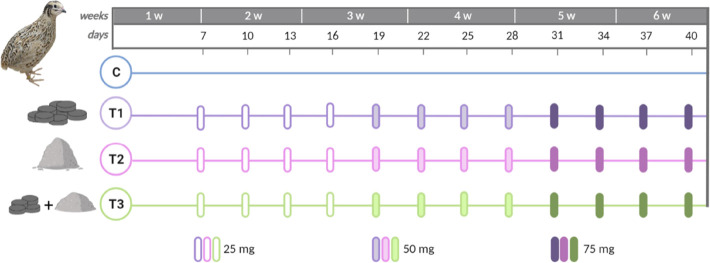
Experimental setup for the exposure of Japanese quail (*Coturnix japonica*) to MP including a control group (C, blue, *n* = 14), treatment 1 exposed to 3 mm MP (T1, purple, *n* = 13), treatment 2 exposed to <125 μm MP (T2, pink, *n* = 14), and treatment 3 exposed to a mixture of 3 mm and <125 μm MP (T3, green, *n* = 14). Each square represents an exposure that was 25 mg the first four doses, 50 mg the next four doses, and 75 mg the last four doses, totaling 600 mg MP per bird.

The design of this experiment consisted of three different treatment groups and a control group (C, Figure 2). The treatment groups were as follows: large MP (T1), small MP (T2), and a mixture of large and small MP (T3). Each group consisted of 14 quails randomly distributed in eight cages with seven quail/cage (1.1 m long × 0.9 m wide × 30 cm high) and two cages/treatment (Section 3, Figure S11). The sample size of this study was selected based on feasibility (husbandry capacity) and ethical reasons (3-R principles) and as a result of prior power analysis. From the initial 56 quails, we ended up with 55 as one quail in T3 needed euthanasia after an accidental trauma.

We started MP exposure at 1 week of age to avoid initial mortality and, afterward, the quail were fed every third day for 5 weeks (Figure 2). With this feeding regime, we aimed to replicate a continuous exposure to plastic during quail’s critical period of development and sexual maturation. The 3-day exposure interval was selected for welfare reasons (i.e., avoid handling the birds every day) and feasibility. Sex was determined when they reached sexual maturity at week 5 of life and included 32 females and 23 males in total (females: C = 9, T1 = 6, T2 = 9, T3 = 8; males: C = 5, T1 = 7, T2 = 5, T3 = 6). The initial body mass of quail at week 1 was on average (±SD) 29.2 ± 5 g.

Both large MP (direct feeding) and small MP (embedded in food pellets) were orally administered to the quail using blunt forceps or spoons, placing the particle or the food pellet on the back of their tongue (Figures S12 and S13). To avoid bias for manipulation and oral administration, control quail were also hand-fed food pellets without MP following the same procedure. The total amount of plastic ingested per bird over the exposure period was 600 mg, either as large MP, small MP, or a mixture of both. The feeding schedule consisted of 12 feeding events, during which the dose of MP was increased twice according to an increase in the size of the quail (Figure 2, Supporting Information Section 4). The dose of MP/g body mass varied between 1.2 and 1.6 during the experiment and was 2.7 at the end of the experiment (Table S1). We could not evaluate individual food consumption as seven quail were housed together, and food was provided *ad libitum*.

The total amount of 600 mg of plastic was chosen based on previously published data.^29−32^ One group of species with the highest levels of plastic ingestion are shearwaters, with a reported average stomach content of 3.2 g MP and an average body mass of 700 g (4.6 mg MP/g body mass).^32^ However, the two experiments performed so far that exposed birds to plastic used lower MP levels, where quail (ca. 300 g body mass) were exposed to 0.15 and 0.3 g of MP^29^ (1 mg/g final body mass) and shearwaters were exposed to 0.4 g of MP (0.6 mg/g final body mass).^31^ Our exposure dose was higher than that used in the previous experiments but still half of the equivalent of maximum environmental exposures reported (2.7 mg/g final BM).

### 2.4 Quail Sampling

Every week during the 6 weeks of the experiment, body mass was measured to the nearest 0.01 g using an electronic balance. At 6 weeks of age (41 days old) and 1 day after the last MP exposure (Figure 2), quail were euthanized by decapitation with scissors. Blood (ca. 3 mL) was immediately sampled using syringes flushed with heparin and stored in heparinized Eppendorf tubes on ice until they could be processed (max. 5 min) to obtain plasma and red blood cells (RBCs) (Supporting Information Section 5). At the same time, small blood samples were collected in capillary tubes and centrifuged (microHt centrifuge: 4000 *g*, 5 min) to record the hematocrit (Ht). Stomachs (including the crop, the proventriculus, and the gizzard) were collected and stored at −20 °C and were later opened to localize and count the 3 mm particles and qualitatively analyze the presence of small MP (Figure S14). Equipment used for the necropsies was thoroughly cleaned with soap and ethanol between individual dissections to avoid cross-contamination.

### 2.5 Laboratory Analysis

#### 2.5.1 Blood-Biochemical Parameters

BCCPs were analyzed in plasma using commercial strips (Reflotron, Roche) and an automated spectrophotometric analyzer (Reflotron, Roche). The targeted analytes consisted of seven different parameters: triglycerides (TG, mmol/L), aspartate aminotransferase (AST, U/L), cholesterol (mmol/L), creatine kinase (CK, U/L), uric acid (μmol/L), amylase (U/L), and glucose (mmol/L). All assays were subjected to daily internal quality controls. To analyze TG and CK, samples were diluted 1:2 and 1:5 respectively with saline solution (NaCl, 9 mg/mL).

#### 2.5.2 Oxidative Stress

The activity of four different antioxidant enzymes [glutathione peroxidase (GPx), glutathione-S-transferase (GST), catalase (CAT), superoxide dismutase (SOD)] and lipid peroxidation estimated by thiobarbituric acid-reactive substances (TBARS) were analyzed in RBC samples. RBCs were homogenized (1:10 w/v, Polytron PT 3000) in buffer (1.15% KCL in 0.01 M PBS with 0.02 M EDTA). The homogenates were centrifuged (4000 *g* for 10 min at 4 °C) and divided into several aliquots and stored at −80 °C until enzyme activity analyses. The activity of CAT, GST, and GPx (nmol min^–1^ mg^–1^ protein) and TBARS (μM malondialdehyde) was determined following Mennillo et al.,^47^ while SOD (U L^–1^) was determined using a commercial assay kit (product no. 706002, Cayman, USA). Total blood protein concentration was measured using the Bradford (1976) method with bovine serum albumin as a standard. All assays were performed in triplicate or duplicate (when not enough sample volume). The intra-assay coefficient of variation (CV) was on average 7.7%, and the interassay CV 11.9% (Supporting Information Section 6, Table S2).

#### 2.5.3 Cytokines

The proinflammatory cytokines interleukin-1β (IL-1 β) and tumor necrosis factor-alpha (TNF-α) were determined in plasma. Because of the presence of lipemia in plasma samples (i.e., visible turbidity in plasma due to the presence of lipoproteins), we first centrifuged the plasma at high speed (4000 *g* for 20 min at 4 °C) to remove the lipid phase.^48^ Chicken IL-1 β and TNF-α ELISA kits (product no. abx250062 for IL-1 β and abx351118 for TNF-α, Abbexa, UK) were used for this experiment, using specific antibodies and standards for each cytokine tested according to the manufacturer’s instructions. All samples were assayed in duplicate in TNF-α and 20 samples in IL-1 β, with an intra-assay CV < 8% for both and an interassay CV < 10% in the case of TNF-α (Supporting Information Section 6, Table S3).

#### 2.5.4 Reproductive Hormones

Testosterone (T) and 17β-estradiol (E_2_) concentrations were analyzed in plasma samples from males and females, respectively. Similar to cytokines (Section 2.5.3), plasma was first centrifuged at high speed to remove the lipid phase followed by the extraction of hormones using a diethyl-ether protocol (detailed in Supporting Information Section 6). The samples were analyzed in duplicate using competitive enzyme immunoassay for T and E_2_ following manufacturer protocols (product no. 582701 for T and 501890 for E_2_, Cayman, USA), with an intra-assay CV ≤ 11% (Table S4).

### 2.6 Statistics

Statistical analyses were carried out using R (version 4.1.2), and figures were created using Graph Pad Prism (version 9). Linear models including treatment group, sex, and cage as fixed factors were used to explore the best model explaining effects of MP ingestion on the different endpoints evaluated. In the case of body mass, a mixed-effect model was used with individual identity as a random effect to account for repeated samples as body mass was measured every week. In all cases, the Akaike’s Information Criterion for small sample sizes (AICc) was used to select the most parsimonious model. The model with the lowest AICc value was selected for further examination. Because the selected models included two or less parameters, we then used two-way analysis of variance to evaluate the significancy of the parameters. When significant, we examined further differences by Dunnett’s post hoc test. Normality of residuals was visually inspected in all final models using histograms, quantile-quantile plots, and boxplots, as well as Shapiro–Wilk tests. When the residuals of the model were not normally distributed, a Kruskal–Wallis model was performed followed by Dunn’s post hoc test. When sex was found to be an influential factor, the parameter was tested again separately for males (*n* = 23) and females (*n* = 32). The hepatosomatic index (HSI) was calculated by dividing the liver mass over the body mass of the quail at the time of euthanasia. The correlations between oxidative stress parameters and cytokines, as well as between reproductive hormones (E_2_, T) and the number of MP found in the stomachs, were assessed through Spearman correlations. Following Muff et al.,^49^ we adopted a gradual language of evidence. Significant levels were set at *P* < 0.05, and *P*-values close to 0.05 (0.05 ≥ *P* < 0.1) were considered as indicative of a trend to avoid missing any biological effects.

## 3 Results and Discussion

### 3.1 Half of the Particles Were Retained in the Stomach

Of the 72 large MP administered to T1 quail throughout the 5 weeks of the experiment, on average (±SD) 33.5 ± 13.1 particles (47%, Table S5) were detected in their stomachs upon the conclusion of the experiment. Quail receiving a mixture of large and small MP (T3) retained slightly more large MP particles in their stomach (65%, 23.3 ± 10 particles, Table S5). We did not observe small MP in the stomach of T2 or T3 quail. In agreement with previous studies in seabirds,^50^ the large MP particles were mostly found in the muscular gizzard, although some were located in the glandular stomach (proventriculus, Figure S14). Ryan^51^ suggested that the plastic size a bird can retain depends not only on the initial size of the ingested plastic, but also on the capacity of the bird species to eliminate it through the gastrointestinal tract. For instance, white-chinned petrels (*Procellaria aequinoctialis*) retain particles ≥1 mm in size,^51^ while in northern fulmars (*Fulmarus glacialis*) this size threshold was higher at ≥2 mm.^50^ Our results suggest that quail of 200–250 g body mass can excrete 3 mm MP. However, because we did not quantify MP in feces, we cannot confirm whether the excretion happened directly through the digestive system or by breaking down the particles to smaller MP.^51^ However, because large MP were not found in the cages of the birds, we believe that pieces were broken down in the stomach as de Souza et al.^30^ recently reported. Importantly, our results indicate large variability in MP retention in the stomachs of individuals of the same species and under the same plastic exposure. Therefore, we suggest that it is the individual, and not only the species, that determines the retention/excretion rate of plastic particles. Factors such as the amount of food consumption, coingestion of smaller plastic sizes, and individual capacity of fragmentation and digestion potentially affect the retention time of plastic particles in the stomach. Because we did not evaluate individual food consumption, we recommend further evaluation of this parameter to address the question of whether MP exposure affects food consumption and whether the latter is related to MP retention.

### 3.2 No Evident Effects on Body Mass or Nutritional Status but Different Growth Rates

**Figure 3:**
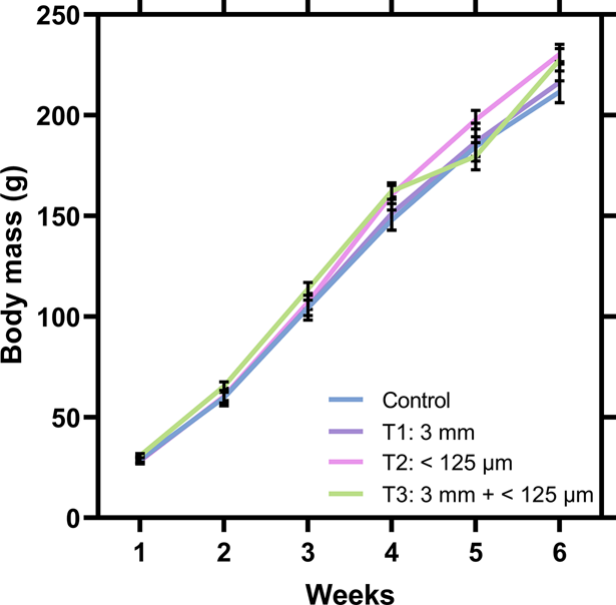
Body mass (g) of Japanese quail (*Coturnix japonica*) measured once per week. The figure compares four treatment groups, control (blue, *n* = 14), T1 (treatment 1: 3 mm particles, purple, *n* = 13), T2 (treatment 2: powder <125 μm, pink, *n* = 14), and T3 (treatment 3: mixture of 3 mm and <125 μm particles, green, *n* = 14). Error bars show standard error.

Exposure to MP affected the growth rate of quail indicated by a significant interaction between week and treatment and showed by different slopes in Figure 3, whereas the final body mass and the nutritional status were not affected by treatment (Supporting Information Section 8).

The growth rate was altered in quail exposed to small MP (T2) and to a mixture of large and small MP (T3) in weeks 4 to 6 (Figure 3, Supporting Information Section 8 & Figure S15). Quail exposed to small MP (T2) grew faster than control quail indicating that small MP induce the growth in quail, even though the final body mass was not significantly higher. The same pattern was observed in quail exposed to a mixture of large and small MP (T3) in week 4, but in week 5 a growth pause was observed. This growth pause could be related to the increase of MP doses in week 4 (Figure 2) and a consequent dietary dilution, as also suggested to occur in male quail in the earlier experiment by Roman et al.^29^ However, and similarly to the former study, we observed that the growth pause resolved from week 5 to 6 and MP exposure does not affect the final body mass after a 5-week exposure. This observed pattern does not directly support the idea of food dilution and contrasts with several experiments performed in other species (e.g., mice^39,52^ and invertebrates^53^) which concluded that MP accumulated in the gastrointestinal tract reduce nutrient uptake and absorption and consequently result in a decrease in body mass. The recent study of de Souza et al.^30^ also showed a decrease in body mass of adult quail exposed to MP. In wild birds, a higher plastic content in the stomachs is often correlated with a lower body mass,^15,32,54^ although this relationship is not always observed.^55,56^ The quail in the present study were able to excrete MP particles and were, thus, probably able to mitigate a food dilution effect. Another plausible explanation is that the growth rate observed in quail exposed to small MP, either alone or in a mixture, could be related to an overcompensation, resulting in an increase of the feeding rate, as suggested in fish.^57^

The HSI is sensitive to environmental pollutants and has been widely used as a measure of the body condition in birds reflecting metabolic energy demands and short-term nutritional status.^58^ Previously, the HSI has been observed to decrease with increasing MP ingestion (e.g., mitten crab *Eriocheir sinensis*(59)). However, in our study, the HSI was significantly influenced by sex but not MP exposure (Figure S16). Furthermore, the average (±SD) Ht was 43 ± 4.6% (Table S6), falling within the reference values for birds^60^ but slightly higher than that reported in quail.^61^ The Ht also differed between sexes, but it was not influenced by MP exposure (Figure S17). Overall, our results indicate no decrease in energy or malabsorption of nutrients in relation to MP exposure.

### 3.3 Microplastics Affect the Hepatic Parameters Cholesterol and AST

BCCPs are useful biomarkers of avian health, reflecting the health status of liver, kidneys, bone, and muscle, as well as energy metabolism.^62^ Yet, only few studies have studied effects of pollutants on BCCPs in birds so far (e.g., raptors,^62,63^ quail^64,65^), and only one study has investigated the relationship with plastic exposure (flesh-footed shearwaters).^32^ In shearwaters, Lavers et al.^32^ found high levels of cholesterol, uric acid, and amylase in birds highly exposed to plastic. Similarly, we found that MP exposure altered cholesterol (χ_3_ = 9.61, *P* = 0.02) and AST levels (*F*_3_ = 2.85, *P* = 0.04), while we did not detect any impact on amylase, uric acid, triglycerides, or glucose (Supporting Information Section 8).

**Figure 4:**
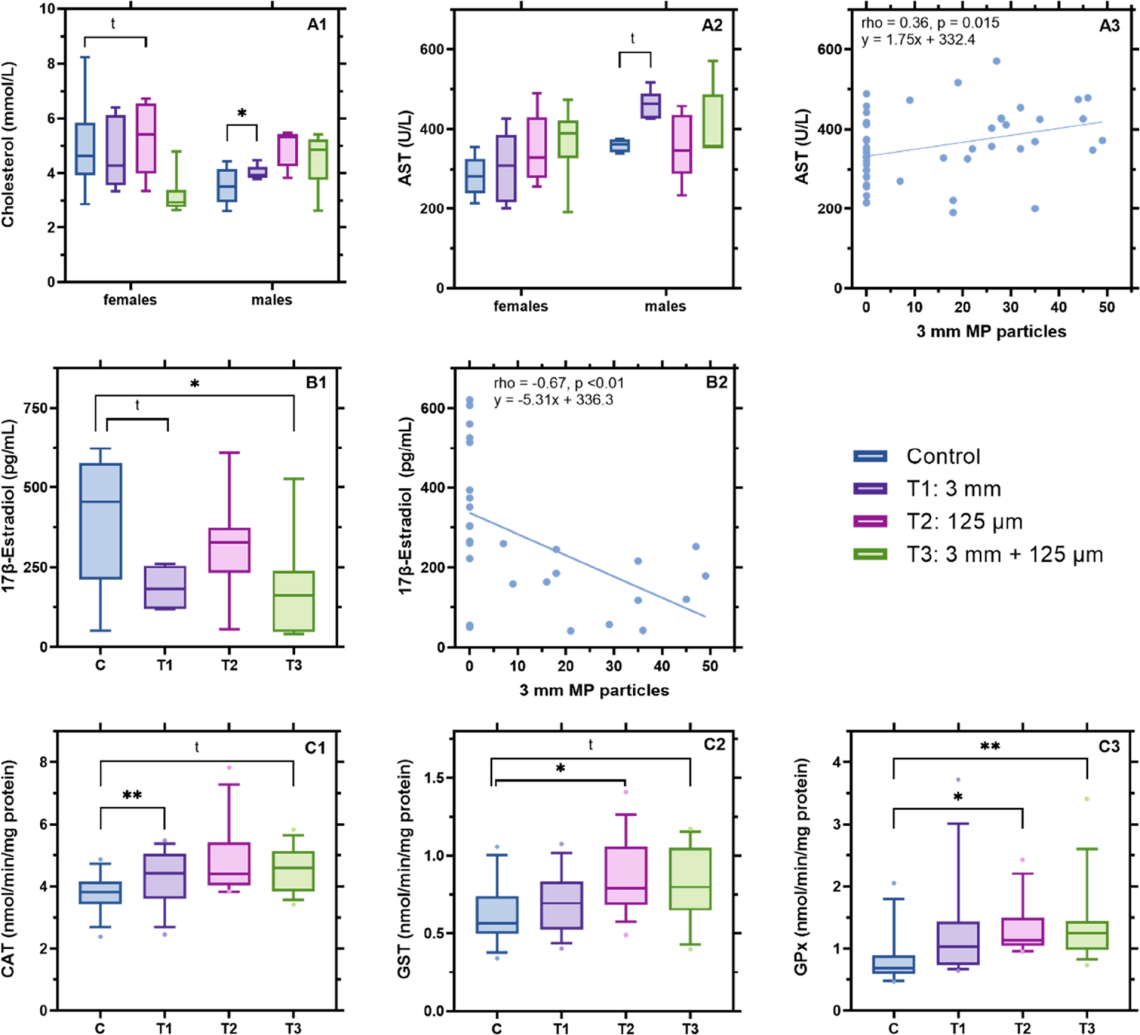
Effects of MP in Japanese quail (*Coturnix japonica*) with regard to (A) BCCPs including cholesterol and AST, (B) female reproductive hormone 17β-estradiol, and (C) oxidative stress parameters including CAT, GST, and GPx. The four treatment groups include the control (blue, *n* = 14), exposure to 3 mm MP (T1, purple, *n* = 14), to <125 μm MP (T2, pink, *n* = 13) and exposure to a mixture of 3 mm and <125 μm MP (T3, green, *n* = 14). Correlation analyses between 3 mm MP particles present in the birds’ stomach and AST levels (A3) or 17β-Estradiol levels (B2) are also shown. Statistical differences (** *P* < 0.01, * *P* < 0.05) and tendencies (^t^ 0.01 > P ≤ 0.05) are shown. To allow for better visualization of the data in graphs B (17β-estradiol), two outliers were removed from the graphs but not the statistical analyses (control female: 1316.7 pg/mL, T2 female: 1495.6 pg/mL).

Cholesterol levels of all groups (Table S6) were within the reference values reported for quail (3–5 mmol/L).^66^ Yet, differences in cholesterol levels were found between treatment groups, and opposite relationships were observed in males and females. Males exposed to small MP (T2) had higher cholesterol levels than control males (Figure 4A1; χ_3_ = 8.80, *P* = 0.03; post hoc *P* = 0.05). These elevated levels of cholesterol are in agreement with the shearwaters study^32^ and could be indicative of metabolic disruption driven by MP^67^ or be related to exposure to plastic-associated chemicals.^68^ Females exposed to the mixture of large and small MP (T3) tended to have lower cholesterol levels than control females (Figure 4A1; *F*_3_ = 5.64, *P* < 0.01; post hoc *P* = 0.09). Females also showed a negative association between cholesterol levels and weight (*F*_1_ = 6.61, *P* = 0.02), but because T3 females had higher body mass than control females (Figure 3), this could rather explain the observed differences.

Quail exposed to large MP, both alone (T1) and in a mixture (T3), tended to have higher AST levels than control quail (Dunnett’s post hoc Test T1 – C *t* = 2.33, *P* = 0.06; T3 – C *t* = 2.29, *P* = 0.07). Sex was also an influential factor, where males had higher AST levels than females (*F*_1_ = 9.97, *P* < 0.01; *t* = 3.16, *P* < 0.01). When analyzed separately by sex, only males showed a trend toward higher AST levels in exposure group T1 in comparison to the control (Figure 4A2; *F*_3_ = 2.92, *P* = 0.06). AST needs to be interpretated in combination with CK to differentiate between liver and muscular damage.^69^ In our case, CK levels had a different pattern compared to AST (Figure S18), and thus muscular damage can be ruled out. Despite differences in AST levels between treatment groups being only a trend, the median values of AST in the quail exposed to large MP (T1 and T3) were higher than 350 U/L (Table S6), which is considered the threshold for hepatic damage in birds.^70^ Elevation of AST in quail was previously reported as a biomarker of liver injury caused by exposure to organic pollutants.^65^ In addition, we found a positive correlation between the exposure to large MP and AST levels (Figure 4A3; rho = 0.36, *P* < 0.01), indicating that quail retaining more MP particles in the stomach might be more susceptible to suffer from hepatic damage.

### 3.4 Large Microplastics Affect Female Reproductive Hormones

Females exposed to large MP, both alone (T1) and in a mixture with small MP (T3), had significantly lower E_2_ levels than control females (Figure 4B1, χ_3_ = 11.91, *P* < 0.01). The difference was larger in quail receiving a mixture of large and small MP (T3: Dunn’s Test *z* = 2.91, *P* = 0.02) than only large MP, which was only a trend (T1: Dunn’s Test *z* = 2.44, *P* = 0.07). There was no significant difference between females exposed only to small MP (T2) and control females (*P* > 0.05, Supporting Information Section 8). These results indicate that MP affect E_2_ levels, seemingly related to the MP size. Moreover, the females retaining a greater number of large MP particles in the stomach had lower levels of E_2_ (Figure 4B2, rho = −0.67, *P* < 0.01). The ability of MP to cause endocrine disruption has been largely discussed;^29,39,71^ however, studies on the effects of MP on bird reproduction are scarce. Only Roman et al.^29^ evaluated reproductive parameters in quail under MP exposure and, unlike our results, did not find any significant hormonal alterations including E_2_ levels. These different results could be explained by the higher amount of MP used in our study, where our results are more in agreement with previous studies in other organisms. For instance, Wang et al.^72^ observed negative effects of MP on female reproductive hormones in fish, but only observed in females’ E_2_ levels. This former study exposed marine medaka to polystyrene 10 μm MP and reported downregulation of transcription of the genes involved in steroidogenesis. In our study, we also only found hormonal effects in females. Male T concentrations did not differ between treatment groups (Supporting Information Section 8 & Figure S19) and were not correlated with the number of large MP in the stomach (*P* > 0.05, Supporting Information Section 8). This lack of significance could be explained by the great variability of T levels within groups (Table S6), which could be indicative of different levels of sexual maturity. It is also likely that MP have sex-specific endocrine disruptive effects as previously reported.^72^ However, the mechanisms of action behind this effect are unknown and require further attention. One important factor to further evaluate is the potential chemical leaching from MP as common chemicals in plastic are known to cause decreased levels of E_2_ (e.g., phthalates in quail^73^).

### 3.5 Small Microplastics Induce Antioxidant Activity but Not Inflammatory Responses

While exposure to MP has been repeatedly reported to induce the formation of reactive oxygen species (ROS) and hence trigger an oxidative stress in fish^37^ and mice,^35^ there is only one study addressing this in birds.^30^ In our study, we observed significant differences between treatment groups in three out of the four antioxidant enzymes analyzed, that is, CAT (Figure 4C1, *F*_3,51_ = 3.93, *P* = 0.01), GST (Figure 4C2, *F*_3,51_ = 3.33, *P* = 0.03), and GPx (Figure 4C3, χ_3_ = 12.86, *P* < 0.01), while SOD remained unaffected (Supporting Information Section 8 & Figure S20). Quail that ingested small MP (T2) had higher activities of CAT (Dunnett’s Test *t* = 3.25, *P* < 0.01), GST (Dunnett’s Test *t* = 2.76, *P* = 0.02), and GPx (Dunn’s Test *z* = −3.11, *P* = 0.01) than control quail. Quail receiving a combination of small and large MP (T3) showed significantly higher GPx levels than control (Dunn’s Test *z* = −3.09, *P* < 0.01) and a trend toward higher levels of CAT (Dunnett’s Test *t* = 2.39, *P* = 0.05) and GST (Dunnett’s Test *t* = 2.26, *P* = 0.07). The increased activities of GST and GPx are in agreement with previous studies in mice^35^ and other organisms,^74^ while CAT activity has been reported both to increase in fish^37^ and decrease in mice^35^ under MP exposure. In a previous study,^30^ CAT levels in quail were observed both to increase and decrease in the same individual exposed to MP depending on the tissues analyzed. CAT and GPx play an important role in protecting tissues against ROS as they breakdown H_2_O_2_ very efficiently^75^ while GST is an indicator of detoxification processes.^76^ In this study, an increase in the antioxidant enzymes possibly reflects an increased production of ROS triggered by MP exposure. However, we did not find evidence of lipid peroxidation, reflected by the nonsignificant differences in TBARS levels between groups (Supporting Information Section 8 & Figure S20). These results suggest that the increase of ROS was likely neutralized by the activation of the antioxidant defense system, as previously observed elsewhere in fish^36^ and quail.^30^

Previous studies also report that MP can trigger inflammatory responses involving the activation of proinflammatory cytokines, for instance, through inducing intestinal dysbacteriosis^38^ or phagocytosis.^77^ At the same time, cytokines can be activated as a response to excessive ROS production,^78^ which can also be triggered by MP exposure. However, we did not find any significant difference between treatment groups either in TNF-α (χ_3_ = 0.90, *P* = 0.83) or in IL-1 β levels (χ_3_ = 1.32, *P* = 0.72). As TNF-α was influenced by sex (χ_1_ = 28.53, *P* < 0.01; Supporting Information Section 8), we investigated treatment differences in TNF-α levels separately in males and females, yet none were significant (Figure S21, both *P* > 0.05). However, in females, a trend toward a negative correlation was found between TNF-α and GPx (rho = −0.32, *P* = 0.07), which indicates decreased TNF-α levels under an increased antioxidant enzyme activity scenario. Imbalance of cytokine production has been reported before (e.g., under heat stress in broilers^79^ and under contaminant exposure in mammals^80^), and a recent review on MP suggested increased cytokine release as a potential pathway related to oxidative stress.^77^ Because we did not find lipid peroxidation, we cannot confirm oxidative stress but rather an activation of the antioxidant enzyme activity. This could have been enough to neutralize the MP effect and to not activate an inflammatory response.

### 3.6 Considerations and Outlook

**Figure 5:**
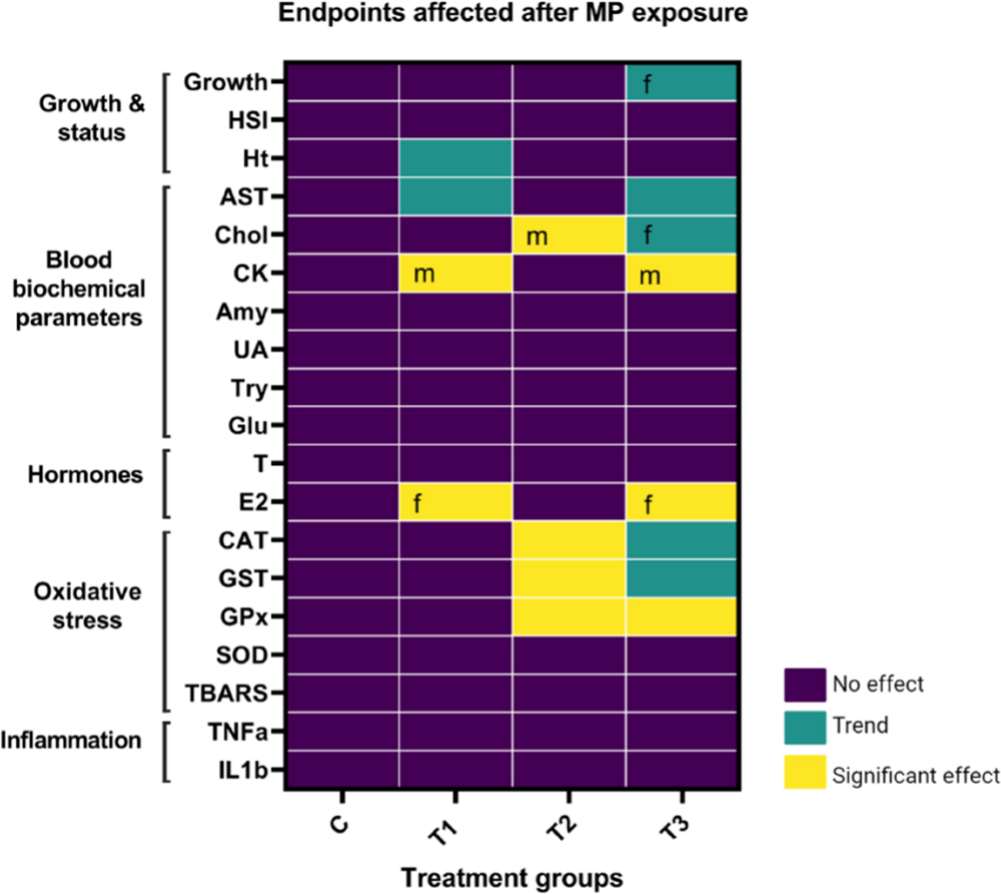
Main endpoints affected after 5 weeks of MP exposure (600 mg) as 3 mm MP (T1), <125 μm MP (T2), and a mixture of both (T3). A control group (C) was also included. The color legend for the heat map indicates no effect (purple), trend (green) and significant effect (yellow). Letters inside the wells indicate sex-specific effects on males (m) or females (f) only.

We used plastic originating from the Norwegian coast and tested two MP sizes separately and as a mixture to increase the environmental relevance of this study. We found that MP ingestion caused sublethal effects which may have far-reaching consequences on bird populations that call for further research. However, we want to highlight the limitations of our study to help improve future research. First, we only evaluated one dose of MP (600 mg) for practical and ethical reasons. This dose was sufficient to observe effects (Figure 5), but it is important that future studies add more dose groups to build dose–response relationships and determine thresholds of MP toxicity. Second, our MP exposure lasted 5 weeks covering the critical period of growth and sexual development of quail. Yet, we could miss important endpoints later in life, such as reproduction impairment or loss in body mass. Long-term exposure studies are required to study these endpoints. Finally, we did not investigate the chemical leaching from the MP we used here. Thus, future studies could address the question of whether the effects of plastic ingestion are caused by physical disruption, chemical toxicity, or a combination of both. We believe that addressing these points may further advance research on MP toxicity in birds.

### 3.7 Summary

This study provides an integrated picture of the effects triggered by MP ingestion in quail, exploring for the first time in birds the effects caused by small-sized MP. We provide evidence that exposure to MP causes an increase of antioxidant enzyme activity, early signs of hepatotoxicity, and decreased E_2_ levels in females (Figure 5), and these responses are dependent on the size of MP and the sex of the birds. In the context of tentative mechanistic pathways (Figure 1), we have differentiated between large and small MP effects highlighting the main endpoints affected by MP exposure (Figure 5) and providing knowledge to advance future research on MP toxicity in birds.

## Supporting Information Available

Additional description of plastic collection and preparation; experimental procedures and set up; laboratory analyses; descriptive data; statistical output; and additional results (PDF)
