# Supplementary material for: Microplastic Ingestion Induces Size-Specific Effects in Japanese Quail
Source: Environ Sci Technol. 2022 Oct 27;56(22):15902–11. doi: 10.1021/acs.est.2c03878 (PMC9671045; doi:10.1021/acs.est.2c03878)
Supplement: Supplementary file 1 — es2c03878_si_001.pdf [file es2c03878_si_001.pdf]

Supporting Information

Microplastic ingestion induces size-specific effects in Japanese quail

Laura Monclús[1*], Eliana McCann Smith[1], Tomasz Maciej Ciesielski[1], Martin Wagner[1], Veerle L.B. Jaspers[1]

[1]Department of Biology, Norwegian University of Science and Technology (NTNU), Høgskoleringen 5, Trondheim 7491, Norway

*Corresponding author: Laura Monclús

E-mail address: laura.monclus@ntnu.no

Full postal address: Department of Biology, Norwegian University of Science and Technology (NTNU), Høgskoleringen 5, Trondheim 7491, Norway

Content (27 pages, 9 sections, 21 Figures, 20 Tables)

1. Plastic 2
   1.1. Collection of plastic 2
   1.2. Plastic cleaning and preparation 2
   1.3. Plastic selection for the experiment 2
   1.4. Plastic preparation for the experiment 3
2. Quail husbandry 6
3. Experimental set up 6
4. Plastic exposure and recovery 7
5. Blood processing 8
6. Laboratory analyses 9
   6.1. Oxidative stress parameters 9
   6.2. Cytokines 10
   6.3. Reproductive hormones 10
   6.4. Plastic particles retention in the stomach 11
7. Descriptive results for all investigated endpoints 12
8. Statistical Output 13
9. Additional Results 21

1. Plastic

1.1 Collection of plastic

Several plastic items were collected from islets in Mausund, Norway (63°52’N, 8°39’E) in March 2020. Fig. S1 shows the environment where the plastic items were collected.

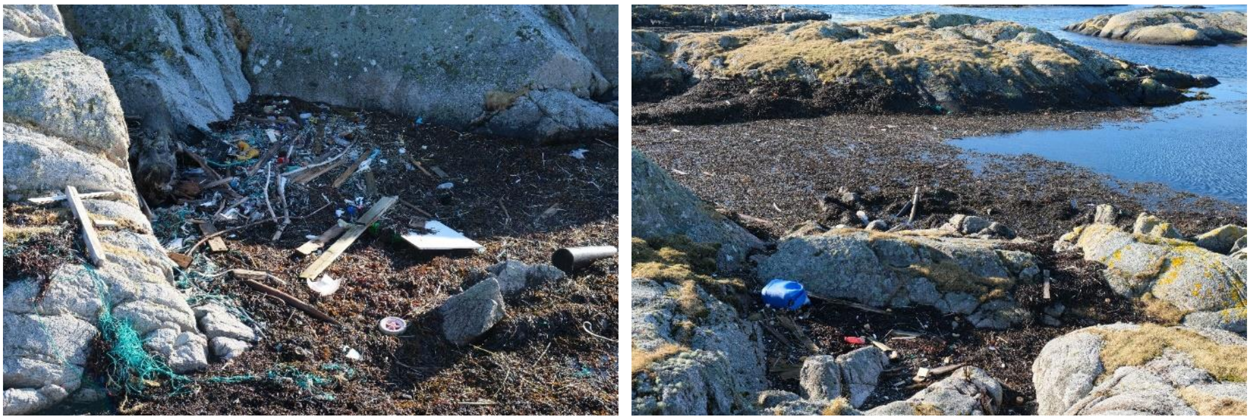

Fig. S1 *Collection of pictures from the field sampling of plastic items in Mausund islets (Trøndelag, Norway) in March 2020.*

1.2 Plastic cleaning and preparation

The collected items (Fig. S2) were gently washed with ultrapure water and further analyzed for polymer type.

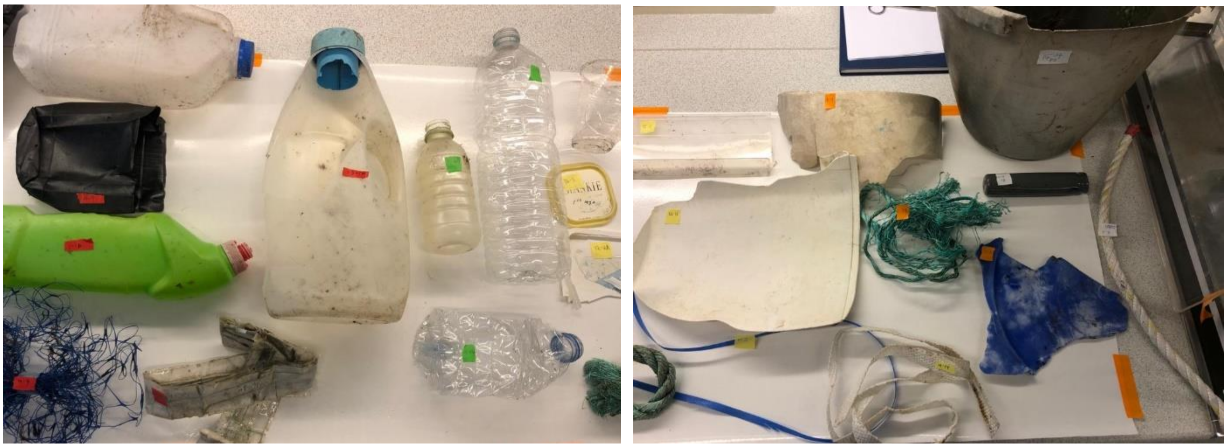

Fig. S2 *Example of plastic items collected, cleaned, and stored in the laboratory for further polymer identification and plastic selection for the experiment.*

1.3 Plastic selection for the experiment

The polymer type was determined using a confocal Raman spectrometer (alpha300 R, WITec) and spectra were confirmed by comparison to the ST-Japan polymer database (L60002). Three items were selected according to unambiguous polymer identification, high abundance in marine environments, and a large volume available for performing the experiment. Fig. S3 – S5 show the three polymer types selected and their RAMAN spectrum.

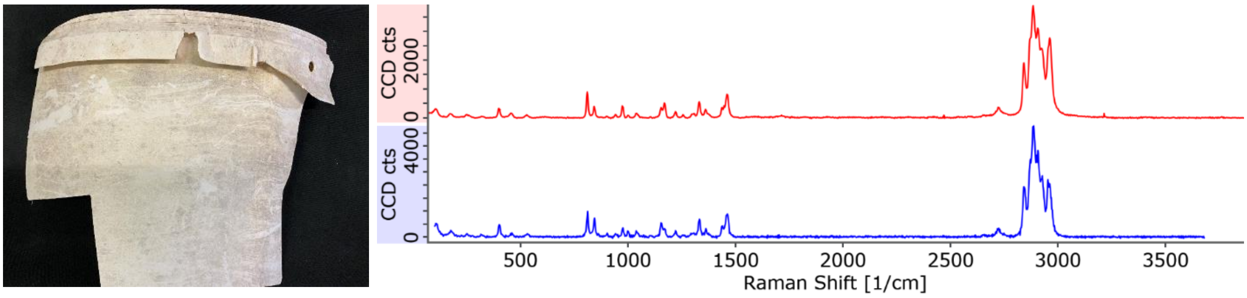

***Fig. S3** Plastic item 1 selected for the experiment and its RAMAN spectrum (red line) aligned with that of the polypropylene from the database (blue line). This item was identified as polypropylene (PP).*

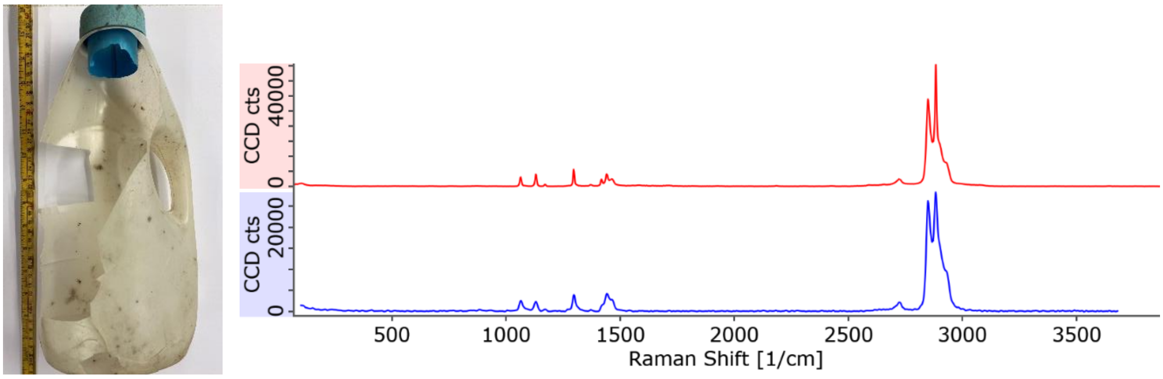

Fig. ***S4*** *Plastic item 2 selected for the experiment and its RAMAN spectrum (red line) aligned with that of the polyethylene from the database (blue line). This item was identified as polyethylene (PE).*

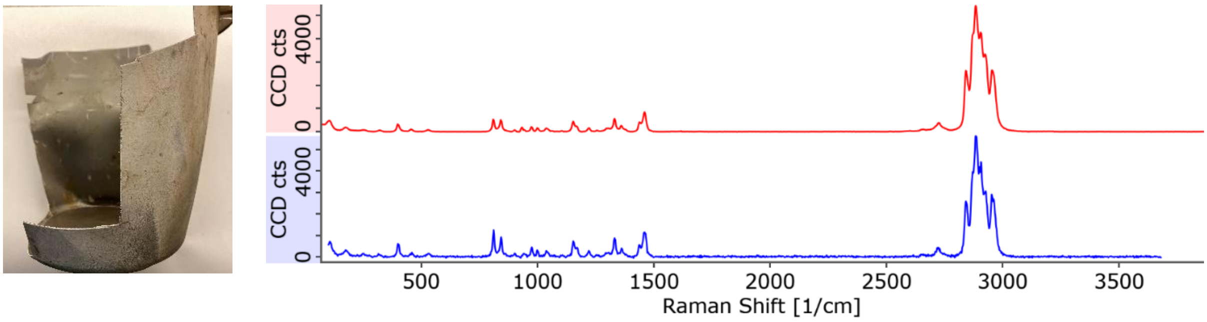

***Fig. S5** Plastic item 3 selected for the experiment and its RAMAN spectrum (red line) aligned with that of the polypropylene from the database (blue line). This item was identified as polypropylene (PP).*

1.4 Plastic preparation for the experiment

Two different size classes were prepared: < 125 µm and 3 mm particles. First, the three plastic items were cut into smaller pieces with scissors. Then, half of the pieces were prepared by punching out 3 mm circular disks using a leather hole-puncher (Fig. S6). The other half of the plastic was cryo-milled to obtain particles < 125 µm (Fig. S7). The cryo-milling was performed through ca. 30 cycles of 2 min immersion in liquid nitrogen followed by 2 min of milling (Retsch Mixer Mill 400) at a frequency of 30 Hz until visual inspection indicated the desired size. The resulting powder was sieved using a vibratory sieve shaker (Retsch AS 200 basic) with a 125 µm sieve, and all MP < 125 µm were collected.

To evaluate the size distribution of the powder (< 125 µm) we used a Coulter counter (Beckam Coulter Miltisizer 4e). Item 1 PP had a mean distribution of 37.78 µm ± 19.0 µm (Fig. S8), Item 2 PE of 33.92 µm ± 11.1 µm (Fig. S9), and Item 3 PP of 43.86 µm ± 21.4 µm (Fig. S10). The overall mean was 38.52 µm ranging from 18.78 to 65.36 µm.

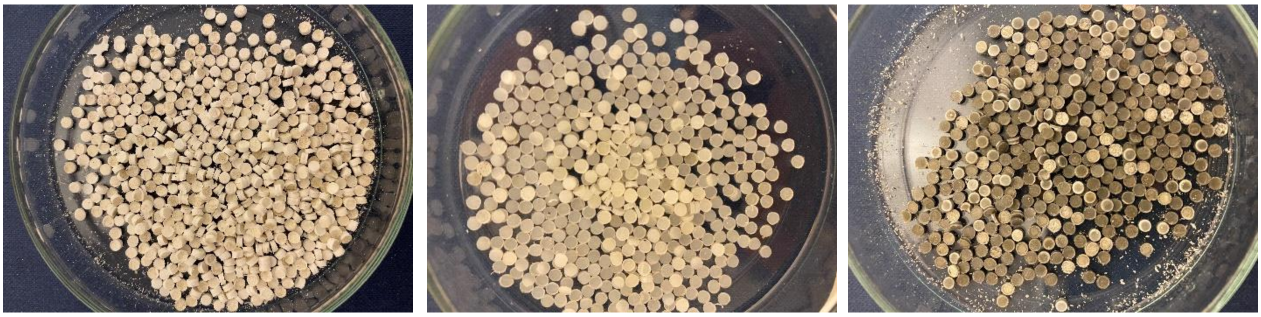

Fig. S6 *Plastic particles prepared in 3 mm size. From left to right; item 1 PP, item 2 PE, item 3 PP.*

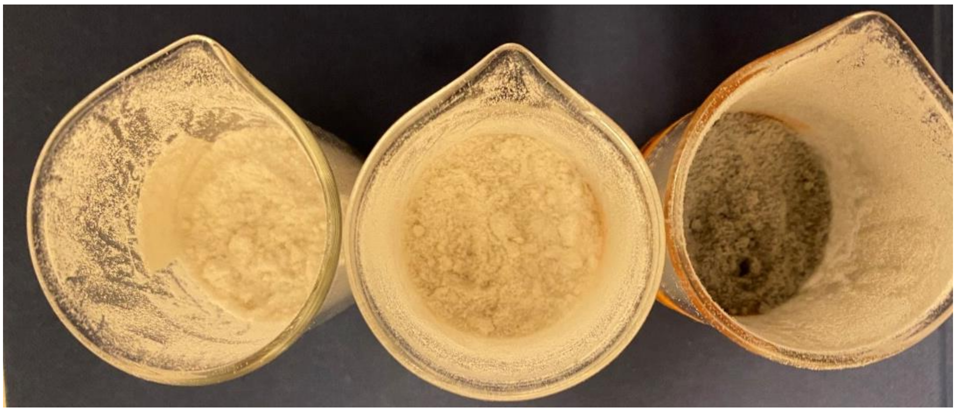

Fig. S7 *Plastic powder prepared < 125 µm size. From left to right; item 1 PP, item 2 PE, item 3 PP.*

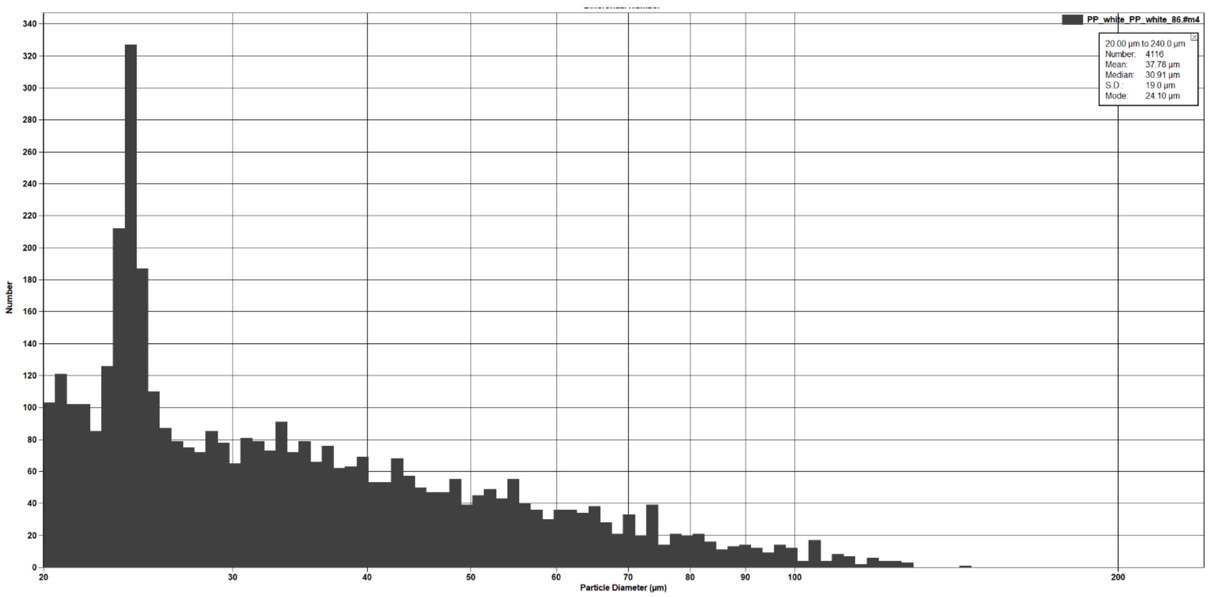

Fig. S8. *Size distribution of MP item 1 PP.*

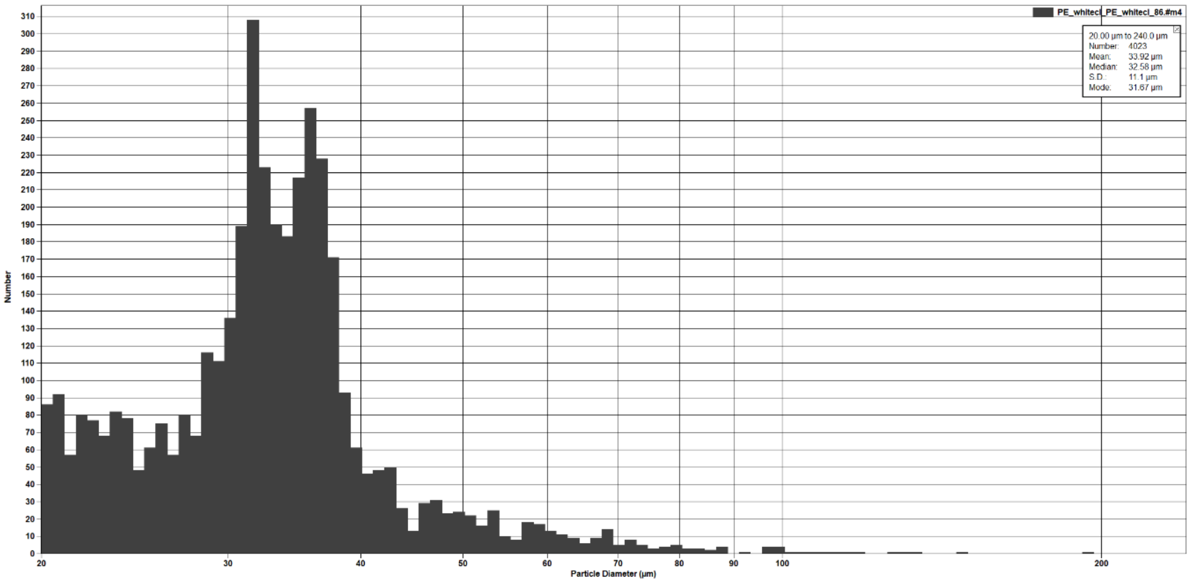

Fig. S9 *Size distribution of MP item 2 PE.*

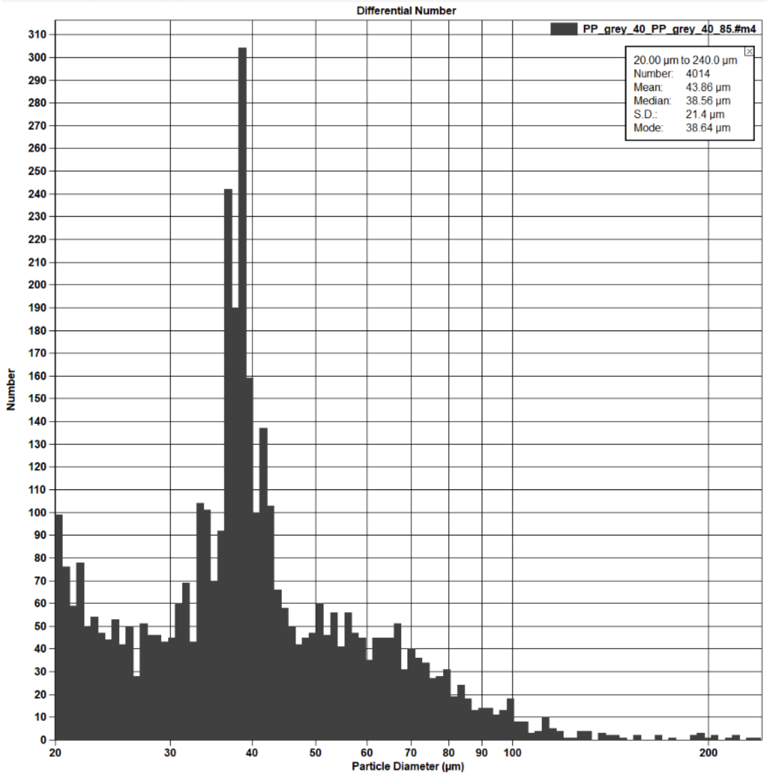

Fig. S10 *Size distribution of MP item 3 PP.*

2. Quail husbandry

In October 2020, 160 quail eggs were purchased from a local farm (Trøndelag, Norway) and kept in incubators (type 180, America A/S, 94 Thisted, Denmark and J. Hemel, Verl, Germany) for 16 days at 38 °C and 50–60 % humidity with automatic egg-turning (90° per hour). The last 2 days before hatching (day 16–18), the egg rotation was stopped, and humidity was increased to 70–75 %. Fifty-six chicks hatched successfully and, once dried (24h post-hatch), they were moved to cages lined with paper and equipped with a cardboard box shelter to increase animal welfare. Eight custom-built wooden cages (1.1 m x 0.9 m) with wire-mesh lids at a height of 30 cm were used. An infrared heat lamp set at 37 °C created a gradient of heat in each cage. The temperature of the heating lamp was decreased by 2–3 °C each week until quail reached 4 weeks of age when the lamps were removed. All cages were maintained in the same room at 22–25 °C with a light-dark cycle of 14–10 h. At week 2, cages were lined with wood chip bedding to encourage natural behaviors such as foraging and dust bathing. Quail were marked with flexible leg bands shortly after hatching for individual identification, which were replaced with larger sizes as the quail grew.

3. Experimental set up

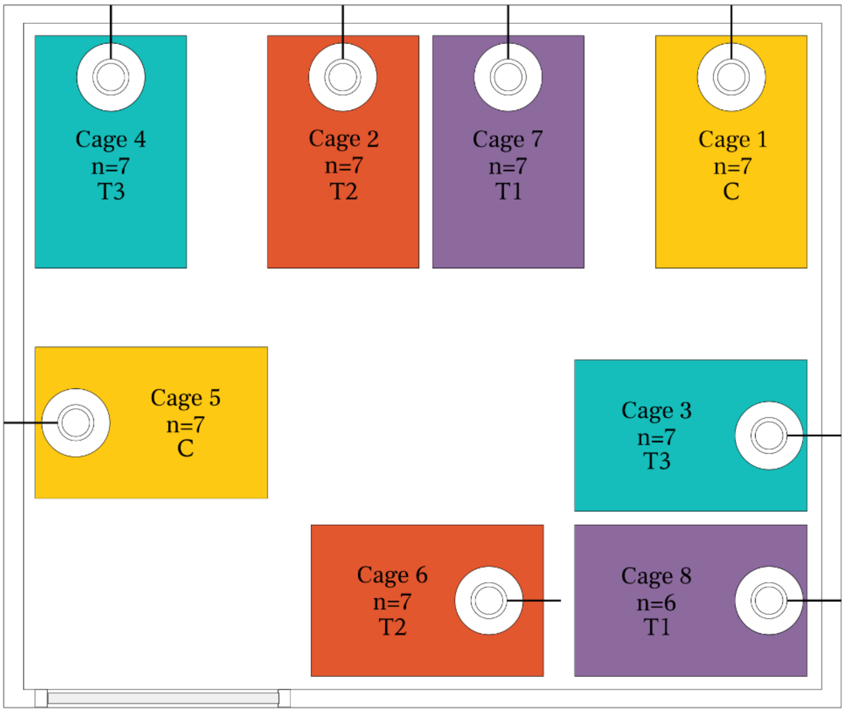

Fig. S11 *Experimental set up. Colors show the different treatments (in yellow control, in purple treatment 1 ingesting 3 mm MP, in red treatment 2 ingesting < 125 µm MP, in blue treatment 3 ingesting both 3 mm and < 125 µm MP). Wooden cages were custom-made with a dimension of 1.1 m x 0.9 m and a wire-mesh lid at a height of 30 cm following recommendations for animal welfare*[1].

4. Plastic exposure and recovery

To administer the plastic powder to the quail, the desired weight of the powder was placed in a ceramic dish where finely ground poultry food and water were added (amount determined visually) in order to form soft pellets of the desired consistency. Finally, to facilitate administration to the quail, the pellets were divided into smaller pellets (Fig. S12).

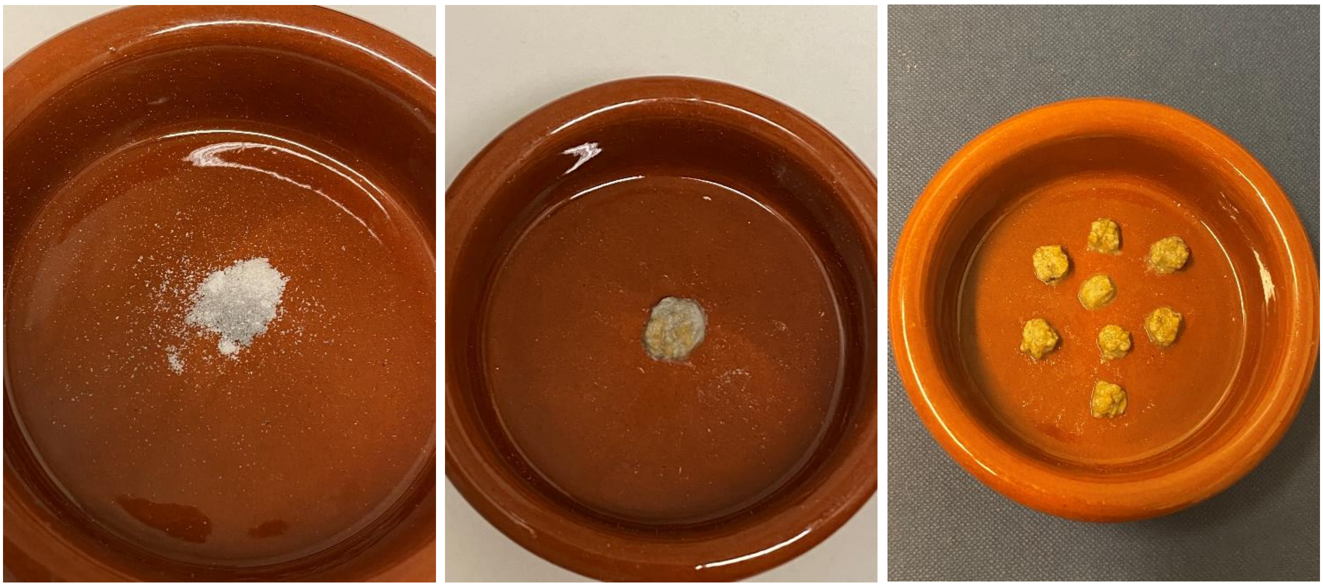

Fig. S12 *The different polymer types in a powder form (left). After grinded food and water were added to create the desired texture of the pellet (middle). The final small pellets created to facilitate oral administration to the quail (right).*

Both 3 mm particles and powder pellets were administered orally to the quail. The 3 mm particles were carefully hand-fed using blunt forceps (Fig. S13), while the powder mixed with food and water was hand-fed using small spoons. To avoid bias for the oral administration, control quail were also captured and hand-fed food pellets following the same procedure.

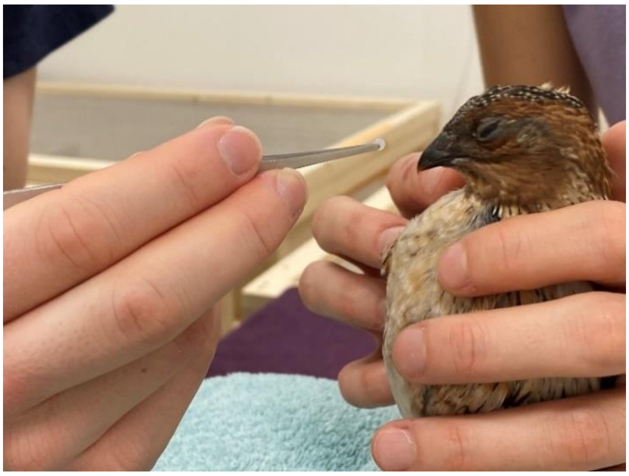

Fig. S13 *Oral administration of a 3 mm particle to a male quail using tweezers*

In total, T1 quail received 72 particles in total per individual (24 of each plastic item), those in T2 received 600 mg of small MP (200 mg of each plastic item), and those in T3 received a mixture of 36 particles (12 of each plastic item) together with 300 mg small MP (100 mg of each plastic item). The dose of MP per g of body mass is shown in Table S1.

Table S1. Dose of MP/g body mass (BM) for each period comprising four feeding events. The final MP/g BM is calculated based on the final BM and total mg MP exposure.

	Period 1	Period 2	Period 3	Final
MP feeding events	4	4	4	12
mg MP given total (feeding events)	100	200	300	600
Average body mass	62/85	156	222	222
Average mg MP/g BM	1.61/1.17	1.28	1.35	2.70
mg MP/g BM T1	1.67/1.21	1.32	1.39	2.76
mg MP/g BM T2	1.66/1.19	1.25	1.30	2.61
mg MP/g BM T3	1.53/1.12	1.23	1.32	2.64

After euthanasia, the stomach tract of the birds was opened to count the remaining 3 mm particles (Fig. S14).

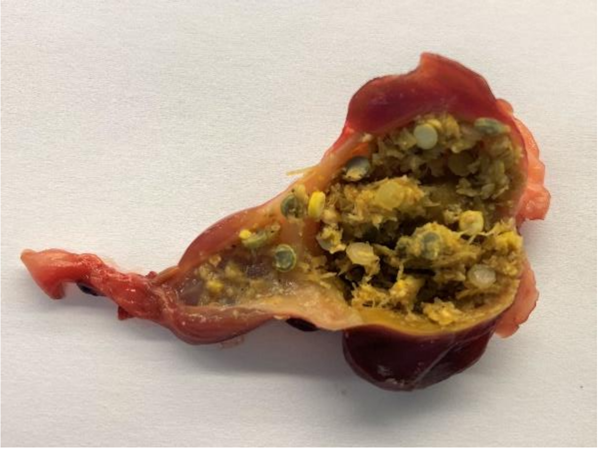

Fig. S14 *Gastrointestinal tract (gizzard and proventriculus) with remaining food and 3 mm particles. The crop cannot be observed in this picture, but it was always opened and evaluated. No particles were found in the crop in any individual.*

5. Blood processing

Blood collected in heparinized Eppendorf tubes was stored on ice until it could be centrifuged (max. 5 min) to separate the plasma and red blood cells (RBC) fractions (4,000 g for 5 min at 4 °C). Plasma was then collected and transferred into a new tube and stored at - 80 °C, while RBC samples were washed with saline solution (NaCl 9 mg/mL) and centrifuged again (4,000 g for 5 min at 4 °C) before storing the supernatant at - 80 °C.

6. Laboratory analyses

6.1 Oxidative stress parameters

The activities of CAT, GST and GPx were determined as described in Mennillo et al.[2]. Briefly, CAT activity was determined based on the peroxidation of methanol in the presence of hydrogen peroxide (H_2O_2). The formaldehyde produced was quantified using a colorimetric assay with 4-amino-3-hydrasino-5-mercapto-1,2,4-triazole (Purpald) as the chromogen, and the absorbance was read at 540 nm. GST was determined using 1-chloro-2,4-dinitrobenzene (CDNB) as a substrate and absorbance was read at 340 nm. An extinction coefficient of 9.6 mM^{-1} cm^{-1} was used to calculate GST activity. The activity of GPx was determined by calculating the rate of NADPH oxidation coupled with glutathione reductase and tert-butyl hydroperoxide (12 mM) and the absorbance was read at 340 nm. The specific activity was determined using an extinction coefficient of 6.22 mM^{-1} cm^{-1}. The activities of CAT, GST and GPx were then divided by the total protein concentration (mg mL^{-1}) determined by the Bradford assay. We used bovine serum albumin (BSA) to prepare the calibration curve and, after adding dye solution (Coomasie blue) to protein samples, the absorption was measured at 595 nm. SOD was quantified using a ®Cayman SOD kit (Ann Arbor, MI, USA) following the instructions of the assay. The plate was read at 450 nm of absorbance. The activity of SOD is given in U L^{-1}. The TBARS assay used the measurement of malondialdehyde (MDA) to construct the standard curve and the absorption was read at 530 nm. All enzymes and protein determinations were done using a Synergy HT microplate reader from Bio-Tek Instruments Inc. (Winnoski, Vermont, USA) for absorbance and fluorescence readings.

All samples were assayed in duplicate or triplicate. Therefore, we used two 96-well plates for each assay. The intra and inter-assay coefficient of variation (CV, mean % ± SE) are determined based on the final concentration for each replicate. The inter-assay CV is calculated based on 2 – 4 samples.

Table S2. Mean % ± SE intra- and inter-assay coefficient of variation (CV).

Assay	Intra-assay CV (%)	Inter-assay CV (%)
Bradford	9.98 ± 1.14	5.30 ± 2.58
CAT	6.77 ± 0.81	8.52 ± 2.12
GPx	10.44 ± 1.43	12.78 ± 2.78
GST	9.53 ± 1.33	6.74 ± 2.54
SOD	6.54 ± 1.53	12.16 ± 2.32
TBARS	2.67 ± 0.75	25.74 ± 0.73*

*In the case of TBARS, a correction to homogenize values from both plates was carried out due to a great difference in the standard curves. Based on three samples that were measured in both plates, a correction of ÷1.5 was applied for all samples from plate 2. After correction, the inter-assay CV was < 5%.

6.2 Cytokines

All samples were assayed in duplicate in TNF-α and 20 samples in IL-1β. We performed the analyses in two 96-well plates for TNF-α and one plate for IL-1β. The intra and inter-assay CV (mean % ± SE) are determined based on the final concentration for each replicate. The inter-assay CV for TNF-α is calculated based on 4 samples.

Table S3. Mean % ± SE intra- and inter-assay coefficient of variation (CV).

Assay	Intra-assay CV (%)	Inter-assay CV (%)
TNF-α	7.13 ± 1.36	9.72 ± 3.09
IL-1β	7.37 ± 1.37	NA

6.3 Reproductive hormones

After high-speed centrifugation to remove the lipid phase, plasma samples were extracted twice with diethyl-ether using 4 times the sample volume (1200 µL), frozen in liquid nitrogen, and the supernatant was subsequently collected. After evaporation of the organic phase overnight, samples were reconstituted with 300 µL of assay buffer and frozen at - 80 °C for analyses.

All samples were assayed in duplicate. The sample size was 23 for testosterone and 32 for 17β-estradiol. We performed one assay for each reproductive hormone. The intra-assay CV (mean % ± SE) are determined based on the final concentration for each replicate.

Table S4. Mean % ± SE intra- and inter-assay coefficient of variation (CV).

Assay	Intra-assay CV (%)
17β-Estradiol (E_2)	10.9 ± 2.92
Testosterone (T)	8.5 ± 1.81

6.4 Plastic particles retention in the stomach

Table S5. Plastic particles recovered from stomach of quail T1 and T3.

Treatment group	Quail	Total plastic particles	% Plastic found/ ingested	Types of polymers		
				Grey	White PP	White PE
T1	1	35	49	10	12	13
	2	38	53	15	12	11
	3	28	39	13	7	8
	4	18	25	5	7	6
	5	7	10	2	1	4
	6	46	64	19	15	12
	7	49	68	17	19	13
	8	19	26	5	7	7
	9	45	63	15	13	17
	10	44	61	12	15	17
	11	28	39	9	8	11
	12	32	44	7	14	11
	13	47	65	16	15	16
	**Mean ± SD**	**33.5 ± 13.1**	**46.6 ± 18.2**			
	**Median**	**35**	**48.6**			
T3	1	26	72	8	8	10
	2	29	81	8	11	10
	3	36	100	13	10	15
	4	22	61	9	6	7
	5	32	89	10	10	12
	6	16	44	8	4	4
	7	29	81	11	8	9
	8	26	72	9	8	7
	9	0	0	0	0	0
	10	18	50	5	4	9
	11	21	58	8	7	6
	12	9	25	5	1	3
	13	27	75	10	8	9
	14	35	97	12	11	12
	**Mean ± SD**	**23.3 ± 10.0**	**64.7 ± 27.8**			
	**Median**	**26**	**72.2**			

7. Descriptive results for all investigated endpoints

Table S6. BCCP levels, oxidative stress parameters, cytokines, reproductive hormones in plasma of Japanese quail (*Coturnix japonica*). Sample size (n), mean ± SE, median and [range] are shown. Treatment (T) groups were as following: T1 (3 mm), T2 (< 125 µm), T3 (mix 3 mm and < 125 µm).

**BCCPs[1]**	**n**	**Glu (mmol L^{-1})**	**Amy (U L^{-1})**	**TG (mmol L^{-1})**	**AST (U L^{-1})**	**Chol (mmol L^{-1})**	**CK (µmol L^{-1})**	**UA (µmol L^{-1})**
**All**	56	19.2 ± 0.3	518 ± 53.7	3.9 ± 0.5	356 ± 11.9	4.4 ± 0.2	3526 ± 190	634 ± 26.2
		18.9 [14.8 – 24.4]	411 [32.5 – 1320]	2.6 [0.8 – 13.7]	351 [190 – 571]	4.2 [2.6 – 8.2]	3255 [267 – 6650]	601 [165 – 1120]
**Control**	14	19.4 ± 0.7	588 ± 101	4.81 ± 1.19	307 ± 15	4.5 ± 0.4	3735 ± 413	605 ± 59.7
		19.5 [15.2 – 24.4]	516 [87.9 – 1320]	3 [0.8 – 13.7]	320 [217 – 375]	4.3 [2.6 – 8.2]	3135 [1760 – 6650]	543 [333 – 1120]
**T1**	13	19.7 ± 0.5	562 ± 157	4 ± 1	385 ± 30.1	4.3 ± 0.3	3349 ± 300	720 ± 51.6
		19.7 [17.3 – 22.8]	420 [196 – 1240]	2.3 [0.8 – 13.3]	426 [200 – 517]	4.1 [3.3 – 6.4]	3245 [1885 – 5850]	661 [479 – 1010]
**T2**	14	18.6 ± 0.6	402 ± 70.9	4.5 ± 0.9	352 ± 21.5	5.2 ± 0.3	3634 ± 522	625 ± 62.4
		18.6 [15.7 – 24.1]	338 [52.5 – 954]	3.5 [0.8 – 11.3]	337 [234 – 489]	5.4 [3.4 – 6.7]	4147 [267 – 6300]	610 [165 – 1060]
**T3**	14	19.2 ± 0.6	563 ± 144	2.2 ± 0.2	382 ± 24.5	3.8 ± 0.3	3372 ± 265	594 ± 31
		19.2 [14.8 – 23.3]	396 [32.5 – 1250]	2.3 [0.8 – 3.4]	369 [190 – 571]	3.3 [2.6 – 5.4]	3270 [1240 – 4590]	591 [336 – 795]

**Oxidative stress[2]**							**Cytokines[3]**	
	**n**	**CAT (nmol min^{-1} mg^{-1})**	**SOD (U L^{-1})**	**GST (nmol min^{-1} mg^{-1})**	**GPx (nmol min^{-1} mg^{-1})**	**TBARS (µm MDA)**	**TNF-α (pg mL^{-1})**	**IL-1 β (pg mL^{-1})**
**All**	56	4.4 ± 0.1	15.9 ± 0.5	0.8 ± 0.03	1.2 ± 0.08	8.1 ± 0.5	69.4 ± 5.06	30 ± 1.1
		4.2 [2.4 – 7.8]	15 [11.1 – 27.7]	0.7 [0.3 – 1.4]	1.1 [0.5 – 3.7]	7.6 [2.7 – 18.4]	62.8 [12.5 – 158]	29.3 [18.6 – 54]
**Control**	14	3.8 ± 0.2	14.4 ± 0.4	0.6 ± 0.05	0.8 ± 0.1	8.02 ± 0.9	78.9 ± 12.1	30.7 ± 1.7
		3.8 [2.4 – 4.9]	14.7 [11.5 – 16.6]	0.6 [0.3 – 1.1]	0.7 [0.5 – 2.1]	7.7 [2.7 – 15.6]	67.2 [18.6 – 152]	30.5 [20.7 – 39]
**T1**	13	4.2 ± 0.3	16 ± 1.3	0.7 ± 0.05	1.3 ± 0.2	7.9 ± 0.9	70.9 ± 11.2	30.6 ± 2.4
		4.4 [2.5 – 5.5]	13.9 [12.4 – 27.7]	0.7 [0.4 – 1.1]	1.03 [0.6 – 3.7]	8.8 [2.9 – 14.7]	67.3 [12.5 – 157]	30.6 [19.5 – 48.2]
**T2**	14	4.9 ± 0.3	16.9 ± 1	0.9 ± 0.07	1.3 ± 0.1	7.9 ± 0.8	63.3 ± 16.9	30.2 ± 2.7
		4.4 [3.8 – 7.8]	15.6 [13.3 – 25.8]	0.8 [0.5 – 1.4]	1.1 [1 – 2.4]	7.5 [3.3 – 12.9]	50.8 [13.4 – 148]	25.7 [18.9 – 54]
**T3**	14	4.6 ± 0.2	16.1 ± 0.9	0.8 ± 0.07	1.4 ± 0.2	8.6 ± 1.3	64.7 ± 6.5	28.5 ± 2.2
		4.6 [3.7 – 5.8]	15.1 [11.1 – 23.6]	0.8 [0.4 – 1.2]	1.3 [0.9 – 3.4]	7 [3 – 18.4]	65.2 [31.4 – 122]	29.2 [18.6 – 46.9]

**Reproductive hormones[4]**	**Males n**	**T (pg mL^{-1})**	**Females n**	**E_2 (pg mL^{-1})**
**All**	23	1660 ± 218	32	365 ± 61.4
		1494 [166 – 4059]		260 [41.4 – 1495]
**Control**	5	1748 ± 410	9	564 ± 132
		1862 [407 – 2881]		514 [50.6 – 1316]
**T1**	7	1641 ± 421	6	186 ± 25.1
		1494 [178 – 3150]		182 [118 – 260]
**T2**	5	1864 ± 697	9	449 ± 140
		1112 [501 – 4059]		352 [55.4 – 1495]
**T3**	6	1440 ± 320	8	181 ± 56.6
		1629 [166 – 2241]		161 [41.4 – 526]

**Nutritional status[5]**	**n**	**HSI**	**Ht (%)**
**All**	56	2.9 ± 0.1	43.1 ± 0.6
		2.8 [1.6 – 5.9]	43 [31 – 53]
**Control**	14	2.5 ± 0.1	41.7 ± 0.9
		2.5 [1.9 – 4.2]	42.5 [36 – 48]
**T1**	13	3.1 ± 0.3	45. ± 1.2
		3 [1.9 – 5.9]	44 [40.4– 53]
**T2**	14	3.2 ± 0.3	42 ± 1.6
		2.9 [1.6 – 5]	42 [31 – 52]
**T3**	14	2.8 ± 0.3	43.6 ± 1
		2.6 [1.7 – 4.9]	44 [36 – 49]

[1] BCCPs: Glu (glucose); Amy (amylase); TG (tryglicerides); AST (asparte aminotransferase); Chol (cholesterol); CK (creatine kinase); UA (uric acid); [2] Oxidative stress parameters: CAT (catalase); SOD (superoxide dismutase); GST (glutathione-S-transferase); GPx (glutathione peroxidase); TBARS (thiobarbituric acid-reactive substances); [3] Cytokines: TNF-α (tumor necrosis factor-alpha); IL-1 β (interleukin-1β)
[4] Reproductive hormones: T (testosterone); E_2 (17-β estradiol); [5]Nutritional status parameters: Hepatosomatic index (HSI); haematocrit (Ht)

8. Statistical Output

The following tables show AICc values for models explaining variation in the studied parameters. The best-supported models (ΔAICc<2), those in bold, were selected for further analysis. Dependent variables included treatment (control, treatment 1, treatment 2, treatment 3), sex, body mass, and cage (C1- C8) distribution. The sample size was 55 quail. As sex was an influential factor in some parameters (HSI, Ht, TG, CK, AST, Chol, Glu, TNF-α), the same models were run again with the sexes separated. Testosterone (T) was only performed for males (n = 23) while 17-β estradiol (E_2) only for females (n = 32).

Table S7. AICc values for models explaining body mass (BM; mg) variation.

Model	df	AICc	Delta	weight
**BM ~ treatment * week + sex + cage + (1|ID)**	**31**	**2501.5**	**0.00**	**0.73**
BM ~ treatment * week + cage + (1|ID)	30	2503.5	1.97	0.73
BM ~ treatment * week + (1|ID)	26	2550.8	49.28	<0.01
BM ~ treatment + week + (1|ID)	11	2634.6	133.1	<0.01
BM ~ week + (1|ID)	8	2646.3	144.8	<0.01
BM ~ 1 + (1|ID)	3	3745.6	1244.1	<0.01

When evaluating differences in body mass, the best model included the interaction between treatment and week, sex, and cage (Table S7). Sex was found not-significant (Table S8), while cage was an influential factor, specially cage 2 (Table S8). To confirm that the interaction between week and treatment was not influenced by cage 2, we rerun the model excluding it and the results obtained were the same (Table S9).

Table S8. Output for models explaining body mass (BM; mg) variation

	Estimate	Std. Error	df	t-value	p-value
Intercept	60.6	5.9	62.7	10.3	<0.001
T1	28.1	8.4	63.5	3.4	<0.01
T2	0.84	8.01	64.5	0.1	0.92
T3	2.88	8.03	64.4	0.4	0.73
Week 3	44.7	3.57	204	12.5	<0.001
Week 4	88.2	3.57	204	24.7	<0.001
Week 5	124.8	3.57	204	34.9	<0.001
Week 6	151.9	3.57	204	42.5	<0.001
Sex M	-1.51	3.89	46	-0.39	0.70
Cage 2	-51.7	7.70	46	-6.71	<0.001
Cage 3	-1.01	7.37	46	-0.14	0.89
Cage 4	5.25	7.43	46	0.71	0.48
Cage 5	-0.71	7.37	46	-0.09	0.92
T1 : week 3	0.24	5.15	204	0.05	0.96
T2 : week 3	2.04	5.05	204	0.40	0.69
T3 : week 3	3.61	5.05	204	0.72	0.48
T1 : week 4	2.89	5.15	204	0.56	0.58

T2 : week 4	11.9	5.05	204	2.37	0.02
T3 : week 4	8.56	5.05	204	1.69	0.09
T1 : week 5	1.93	5.15	204	0.37	0.71
T2 : week 5	12.6	5.05	204	2.49	0.01
T3 : week 5	-10.6	5.05	204	-2.09	0.04
T1 : week 6	4.49	5.15	204	0.87	0.38
T2 : week 6	17.9	5.05	204	3.55	<0.001
T3 : week 6	10.1	5.05	204	1.99	0.04

Table S9. Output for models explaining body mass (BM; mg) variation excluding cage 2

	Estimate	Std. Error	df	t-value	p-value
Intercept	60.3	6.23	51.6	9.69	<0.001
T1	16.3	9.09	60.2	1.79	0.08
T2	0.84	8.38	53.1	0.10	0.92
T3	2.99	8.40	52.9	0.36	0.72
Week 3	44.7	3.43	176	13.0	<0.001
Week 4	88.3	3.43	176	25.8	<0.001
Week 5	124.8	3.43	176	36.4	<0.001
Week 6	151.9	3.43	176	44.3	<0.001
Sex M	-0.78	4.45	40	-0.17	0.86
Cage 3	-0.91	7.82	40	-0.12	0.91
Cage 4	5.04	7.89	40	0.64	0.53
Cage 5	-0.60	7.82	40	-0.08	0.94
T1 : week 3	8.26	6.26	176	1.32	0.19
T2 : week 3	2.04	4.85	176	0.42	0.68
T3 : week 3	3.61	4.85	176	0.75	0.46
T1 : week 4	16.5	6.26	176	2.63	<0.01
T2 : week 4	11.9	4.85	176	2.47	0.01
T3 : week 4	8.56	4.85	176	1.77	0.07
T1 : week 5	19.9	6.26	176	3.19	<0.01
T2 : week 5	12.6	4.85	176	2.60	0.01
T3 : week 5	-10.6	4.84	176	-2.18	0.03
T1 : week 6	22.9	6.25	176	3.67	<0.001
T2 : week 6	17.9	4.85	176	3.71	<0.001
T3 : week 6	10.1	4.85	176	2.08	0.03

Since we found a significant interaction between week and treatment, resulting in different slopes in the growth of the quail, we also investigated potential statistical differences in the body mass of quail each week per separate (Table S10).

Table S10. Output for models explaining body mass (BM, mg) variation. Models were selected based on AICc contrast.

	**Selected model**	**ANOVA output**	**Dunnett test**
w1	BM ~ treatment + cage	Treatment: F_3 = 1.19; P = 0.32	
		Cage: F_4= 7.59; P < 0.01	**C2 – C1 P = 0.02**

			C3 – C1 P > 0.05
			C4 – C1 P > 0.05
			C5 – C1 P > 0.05
			C6 – C1 P > 0.05
			C7 – C1 P > 0.05
			C8 – C1 P > 0.05
w2	BM ~ treatment + cage	Treatment: F_3 = 1.59; *P* = 0.20	
		Cage: F_4 = 11.10; *P* < 0.01	**C2 – C1 P = 0.03**
			C3 – C1 P > 0.05
			C4 – C1 P > 0.05
			C5 – C1 P > 0.05
			C6 – C1 P > 0.05
			C7 – C1 P > 0.05
			**C8 – C1 P < 0.01**
w3	BM ~ treatment + cage	Treatment: F_3 = 1.69; *P* = 0.18	
		Cage: F_4= 10.88; *P* < 0.01	**C2 – C1 P = 0.03**
			C3 – C1 P > 0.05
			C4 – C1 P > 0.05
			C5 – C1 P > 0.05
			C6 – C1 P > 0.05
			C7 – C1 P > 0.05
			**C8 – C1 P < 0.01**
w4	BM ~ treatment + cage	Treatment: F_3 = 2.63; *P* = 0.06	T1 – cnt P > 0.05
			T2 – cnt P > 0.05
			T3 – cnt P > 0.05
		Cage: F_4 = 9.67; *P* < 0.01	**C2 – C1 P = 0.06**
			C3 – C1 P > 0.05
			C4 – C1 P > 0.05
			C5 – C1 P > 0.05
			C6 – C1 P > 0.05
			C7 – C1 P > 0.05
			**C8 – C1 P < 0.01**
w5	BM ~ treatment + cage	Treatment: F_3 = 2.88; *P* = 0.05	T1 – cnt P > 0.05
			T2 – cnt P > 0.05
			T3 – cnt P > 0.05
		Cage: F_4 = 15.28; *P* < 0.01	**C2 – C1 P = 0.01**
			C3 – C1 P > 0.05
			C4 – C1 P > 0.05
			C5 – C1 P > 0.05
			C6 – C1 P > 0.05
			**C7 – C1 P = 0.05**
			**C8 – C1 P < 0.01**
w6	BM ~ treatment + cage + sex	Treatment: F_3 = 2.94; *P* = 0.04	T1 – cnt P > 0.05
			T2 – cnt P > 0.05
			T3 – cnt P > 0.05
		Sex: F_1 = 1.31; *P* = 0.26	
		Cage: F_4 = 9.66; *P* < 0.01	C2 – C1 P > 0.05
			C3 – C1 P > 0.05
			C4 – C1 P > 0.05
			C5 – C1 P > 0.05
			C6 – C1 P > 0.05
			C7 – C1 P > 0.05
			**C8 – C1 P < 0.01**

Table S11. AICc values for models explaining variation in HSI (Hepatosomatic Index).

	Model	df	AICc	Delta	weight
HSI all	**HSI ~ treatment + sex**	**6**	**-363.8**	**0.00**	**0.941**
	HSI ~ treatment * sex	9	-358.2	5.61	0.057
	HSI ~ treatment * sex + cage	13	-351.3	12.50	<0.01
	HSI ~ 1	2	-340.8	22.99	<0.01
	HSI ~ treatment	5	-336.4	27.42	<0.01
HSI males	**HSI ~ 1**	**2**	**-166.9**	**0.00**	**0.637**
	**HSI ~ treatment**	**5**	**-165.8**	**1.13**	**0.363**
	HSI ~ treatment + cage	9	-148.2	18.68	<0.01
HSI females	**HSI ~ 1**	**2**	**-201.6**	**0.00**	**0.857**
	HSI ~ treatment	5	-197.7	3.97	0.118
	HSI ~ treatment + cage	9	-194.5	7.09	0.025

Table S12. Output for models explaining HSI variation.

	Selected model	ANOVA output
HSI all	HSI ~ treatment + sex	Sex: $F_{1,50}$ = 34.16; P < 0.001<br>Treatment: F_3 = 1.37; P = 0.26
HSI male	HSI ~ treatment	Treatment: $F_{3,19}$ = 2.56; P = 0.08

Table S13. AICc values for models explaining variation in Ht (hematocrit %).

	Model	df	AICc	Delta	weight
Ht all	**Ht ~ sex**	**3**	**301.4**	**0.00**	**0.574**
	**Ht ~ sex + treatment + weight**	**7**	**303.1**	**1.74**	**0.240**
	Ht ~ sex + treatment	6	303.7	2.32	0.180
	Ht ~ sex + treatment + weight + cage	11	310.7	9.32	<0.01
	Ht ~ sex * treatment + weight + cage	14	318.2	16.84	<0.01
	Ht ~ 1	2	327.5	26.08	<0.01
Ht males	**Ht ~ 1**	**2**	**130.8**	**0.00**	**0.934**
	Ht ~ treatment	5	136.9	6.12	0.044
	Ht ~ treatment + weight	6	138.3	7.50	0.02
	Ht ~ treatment + weight + cage	10	157.3	26.47	<0.01
Ht females	**Ht ~ 1**	**2**	**172.8**	**0.00**	**0.769**
	Ht ~ treatment	5	175.8	3.01	0.170
	Ht ~ treatment + weight	6	177.9	5.14	0.06
	Ht ~ treatment + weight + cage	10	184.9	12.11	<0.01

Table S14. Output for models explaining Ht variation.

	Selected model	ANOVA output
Ht all	Ht ~ sex + treatment + weight	Sex: F_1 = 32.35, P < 0.01<br>Body weight: F_1 = 2.94, P = 0.09<br>Treatment: F_3 = 3.07, P = 0.03

We found that T1 quail tended to show higher Ht than control quail (post-hoc t = 2.12, P = 0.09), but this could instead be due to arbitrary slight dehydration[3] than an effect of MP. When analyzed separately by sex, no further differences were found (Table S13).

Table S15. AICc values for models explaining variation in BCCPs. Note: BM = body mass, TG = triglycerides, CK = creatine kinase, AST = aspartate aminotransferase, UA = uric acid, Chol = cholesterol, Amy = amylase, Glu = glucose. Residuals for TG, CK, AST and UA were normally distributed, while residuals for Chol, Amy and Glu were not normally distributed.

	Model	df	AICc	Delta	weight
TG	**TG ~ treatment + sex + BM**	**7**	**281.6**	**0.00**	**0.859**
	TG ~ treatment + sex	6	285.5	3.84	0.126
	TG ~ treatment + sex + BM + cage	11	289.9	8.27	0.01
	TG ~ 1	2	194.8	13.14	<0.01
	TG ~ treatment	5	296.8	15.13	<0.01
TG males	**TG ~ BM**	**3**	**58.0**	**0.00**	**0.53**
	**TG ~ 1**	**2**	**58.3**	**0.28**	**0.46**
	TG ~ treatment + BM	6	65.8	7.82	0.01
	TG ~ treatment + BM + cage	10	81.3	23.26	<0.01
TG females	**TG ~ BM**	**3**	**173.7**	**0.00**	**0.86**
	TG ~ BM + treatment	6	177.5	3.86	0.13
	TG ~ 1	2	182.3	8.59	0.01
	TG ~ BM + treatment + cage	10	188.5	14.77	<0.01
CK	**CK ~ sex**	**3**	**952.5**	**0.00**	**0.868**
	CK ~ 1	2	957.8	5.34	0.06
	CK ~ treatment + sex	6	958.0	5.49	0.06
	CK ~ treatment + sex + BM	7	960.5	8.00	0.02
	CK ~ treatment + sex + BM + cage	11	968.0	15.55	<0.01
CK males	**CK ~ treatment**	**5**	**395.6**	**0.00**	**0.66**
	CK ~ 1	2	397.9	2.29	0.21
	CK ~ treatment + BM	6	398.9	3.35	0.12
	CK ~ treatment + BM + cage	10	415.1	19.54	<0.01
CK females	**CK ~ 1**	**2**	**557.0**	**0.00**	**0.95**
	CK ~ treatment	5	563.8	6.85	0.03
	CK ~ treatment + BM	6	565.2	8.21	0.01
	CK ~ treatment + BM + cage	10	576.9	19.92	<0.01
AST	**AST ~ treatment + sex**	**6**	**607.1**	**0.00**	**0.577**
	**AST ~ treatment + sex + BM**	**7**	**697.9**	**0.79**	**0.389**
	AST ~ treatment	5	614.6	7.45	0.01
	AST ~ 1	2	614.8	7.66	0.01
	AST ~ treatment + sex + BM + cage	11	615.9	8.79	<0.01
AST males	**AST ~ 1**	**2**	**233.8**	**0.00**	**0.57**
	**AST ~ treatment**	**5**	**234.6**	**0.85**	**0.38**
	AST ~ treatment + BM	6	238.6	4.81	0.05
	AST ~ treatment + BM + cage	10	256.1	22.37	<0.01
AST females	**AST ~ 1**	**2**	**375.3**	**0.00**	**0.58**
	**AST ~ treatment**	**5**	**376.9**	**1.66**	**0.26**
	AST ~ treatment + BM	6	377.9	2.69	0.15

	AST ~ treatment + BM + cage	10	390.0	14.73	<0.01
UA	**UA ~ 1**	**2**	**740**	**0.00**	**0.766**
	UA ~ treatment	5	743.4	3.40	0.140
	UA ~ treatment + sex	6	744.7	4.69	0.07
	UA ~ treatment + sex + BM	7	747.3	7.32	0.02
	UA ~ treatment + sex + BM + cage	11	755.9	15.93	<0.01
Chol	**Chol ~ treatment + BM**	**6**	**176.9**	**0.00**	**0.547**
	**Chol ~ treatment + BM + sex**	**7**	**178.0**	**1.09**	**0.317**
	Chol ~ treatment	5	180.1	3.20	0.110
	Chol ~ 1	2	183.5	6.60	0.02
	Chol ~ treatment + BM + sex + cage	11	186.1	9.22	< 0.01
Chol males	**Chol ~ treatment**	**5**	**58.3**	**0.00**	**0.64**
	**Chol ~ 1**	**2**	**60.1**	**1.79**	**0.26**
	Chol ~ treatment + BM	6	61.9	3.63	0.10
	Chol ~ treatment + BM + cage	10	80.9	22.55	<0.001
Chol females	**Chol ~ treatment + BM**	**6**	**109.9**	**0.00**	**0.87**
	Chol ~ treatment	5	113.9	3.95	0.12
	Chol ~ 1	2	119.0	9.10	<0.01
	Chol ~ treatment + BM + cage	10	123.2	13.27	<0.01
Amy	**Amy ~ weight**	**3**	**628.7**	**0.00**	**0.680**
	**Amy ~ 1**	**2**	**630.5**	**1.81**	**0.275**
	Amy ~ treatment + BM	6	634.9	6.20	0.03
	Amy ~ treatment + BM + sex	7	636.4	7.67	0.02
	Amy ~ treatment + BM + sex + cage	10	644.8	16.11	< 0.01
Glu	**Glu ~ sex**	**3**	**236.1**	**0.00**	**0.93**
	Glu ~ sex + treatment	6	242.1	6.03	0.05
	Glu ~ sex + treatment + cage	10	243.7	7.60	0.02
	Glu ~ sex + treatment + cage + BM	11	246.8	10.73	<0.01
	Glu ~ 1	2	249.7	13.63	<0.01
Glu males	**Glu ~ 1**	**2**	**90.1**	**0.00**	**0.92**
	Glu ~ treatment	**5**	**95.0**	**4.88**	**0.08**
	Glu ~ treatment + cage	9	108.7	18.59	<0.01
	Glu ~ treatment + cage + BM	10	109.2	19.06	<0.01
Glu females	**Glu ~ 1**	**2**	**145.5**	**0.00**	**0.95**
	Glu ~ treatment	5	151.9	6.41	0.04
	Glu ~ treatment + BM	9	154.2	8.69	0.01
	Glu ~ treatment + BM + cage	10	158.2	12.74	<0.01

Similar to AST (detailed in the main Manuscript), CK levels were also higher in males than females (F_1 = 7.83, P < 0.01; t = 2.79, P < 0.01; Fig S18), and when analyzed separately by sex, only males showed a difference between treatment groups (F_3 = 3.84, P = 0.03) with T2 and T3 males showing lower CK levels than control males (both P < 0.05). Because the pattern of CK was not the same as AST, we could rule out a possible muscular damage.

Table S16. AICc values for models explaining variation in oxidative stress parameters. Note: CAT = catalase, GST = glutathione-S-transferase, TBARS = thiobarbituric acid-reactive substances, SOD = superoxide dismutase, GPx = glutathione peroxidase. Residuals for CAT, GST and TBARS were normally distributed, while residuals for SOD and GPx were not normally distributed.

	Model	df	AICc	Delta	weight
CAT	**CAT ~ treatment**	**5**	**152.9**	**0.00**	**0.792**
	CAT ~ treatment + cage	9	157.1	4.19	0.098
	CAT ~ 1	2	157.3	4.45	0.086
	CAT ~ treatment + cage + sex	10	159.8	6.92	0.025
GST	**GST ~ treatment**	**5**	**-1.4**	**0.00**	**0.692**
	GST ~ 1	2	1.4	2.84	0.167
	GST ~ treatment + cage	9	2.2	3.65	0.112
	GST ~ treatment + cage + sex	10	5.0	6.37	0.029
TBARS	**TBARS ~ 1**	**2**	**287.0**	**0.00**	**0.942**
	TBARS ~ treatment	5	293.7	6.72	0.033
	TBARS ~ treatment + cage	9	294.9	7.98	0.017
	TBARS ~ treatment + cage + sex	10	296.5	9.55	<0.01
**SOD**	**SOD ~ 1**	**2**	**295.2**	**0.00**	**0.712**
	SOD ~ treatment	5	298.3	3.04	0.156
	SOD ~ treatment + cage	9	299.9	4.64	0.070
	SOD ~ treatment + cage + sex	10	300.1	4.86	0.063
GPx	**GPx ~ 1**	**2**	**107.6**	**0.00**	**0.512**
	**GPx ~ treatment**	**4**	**108.0**	**0.40**	**1.419**
	GPx ~ treatment + cage	9	112.3	4.72	0.048
	GPx ~ treatment + cage + sex	10	113.9	6.38	0.021

Table S17. Output for models explaining variation for CAT, GST and GPx.

	Selected model	ANOVA / Kruskal-Wallis output	Dunnett / Dunn's test
CAT	CAT ~ treatment	Treatment: $F_{3,51} = 3.93$, $P = 0.01$	C – T1: P > 0.05<br>**C – T2: $t = 3.25$, $P < 0.01$**<br>**C – T3: $t = 2.39$, $P = 0.05$**
GST	GST ~ treatment	Treatment: $F_{3,51} = 3.33$, $P = 0.03$	C – T1: P > 0.05<br>**C – T2: $t = 2.76$, $P = 0.02$**<br>**C – T3: $t = 2.26$, $P = 0.07$**
GPx	GPx ~ treatment	Treatment: $\chi_3 = 12.86$, $P < 0.01$	C – T1: P > 0.05<br>**C – T2: $z = -3.11$, $P = 0.01$**<br>**C – T3: $z = -3.09$, $P < 0.01$**

Table S18. AICc values for models explaining variation in cytokines.

	Model	df	AICc	Delta	weight
TNF-α	**TNF-α ~ treatment + sex**	**6**	**530.3**	**0.00**	**0.99**
	TNF-α ~ treatment + sex + cage	10	539.4	9.15	0.01
	TNF-α ~ treatment + sex + cage + weight	13	546.1	15.8	<0.01
	TNF-α ~ 1	2	559.0	28.8	<0.01

	TNF-α ~ treatment	5	564.5	34.2	<0.01
TNF-α males	**TNF-α ~ 1**	**2**	**222.9**	**0.00**	**0.89**
	TNF-α ~ treatment	5	227.3	4.43	0.09
	TNF-α ~ treatment + weight	6	130.8	7.90	0.02
	TNF-α ~ treatment + weight + cage	10	246.6	23.65	<0.01
TNF-α females	**TNF-α ~ 1**	**2**	**306.8**	**0.00**	**0.92**
	TNF-α ~ treatment	5	312.2	5.37	0.06
	TNF-α ~ treatment + weight	6	314.7	7.85	0.02
	TNF-α ~ treatment + weight + cage	10	320.0	13.15	<0.01
IL-1β	**IL-1β ~ 1**	**2**	**353.6**	**0.00**	**0.95**
	IL-1β ~ treatment	5	360.0	6.48	0.04
	IL-1β ~ treatment + sex	6	362.1	8.57	0.01
	IL-1β ~ treatment + sex + cage	10	372.5	18.95	<0.01
	IL-1β ~ treatment + sex + cage + weight	13	382.8	29.20	<0.01

Table S19. AICc values for models explaining variation in E_2 (17-β Estradiol) concentrations.

	Model	df	AICc	Delta	weight
E_2	**E_2 ~ treatment**	**5**	**467.9**	**0.00**	**0.59**
	**E_2 ~ 1**	**2**	**468.6**	**0.71**	**0.41**
	E_2 ~ treatment + weight	9	478.5	10.56	<0.01
	E_2 ~ treatment + weight + cage	10	482.1	14.23	<0.01
T	**T ~ 1**	**2**	**388.7**	**0.00**	**0.98**
	T ~ treatment	5	397.1	8.41	0.02
	T ~ treatment + weight	6	399.7	11.02	<0.01
	T ~ treatment + weight + cage	10	419.1	30.31	<0.01

Table S20. Output for the Dunn's Test for E_2 variation in females.

**Comparison**	**Z-value**	**P-adj (Holm method)**
Control – T1	2.44	**0.07**
Control – T2	0.65	0.76
T1 – T2	-1.85	0.18
Control – T3	2.91	**0.02**
T1 – T3	0.24	0.81
T2 – T3	2.28	**0.08**

9. Additional Results

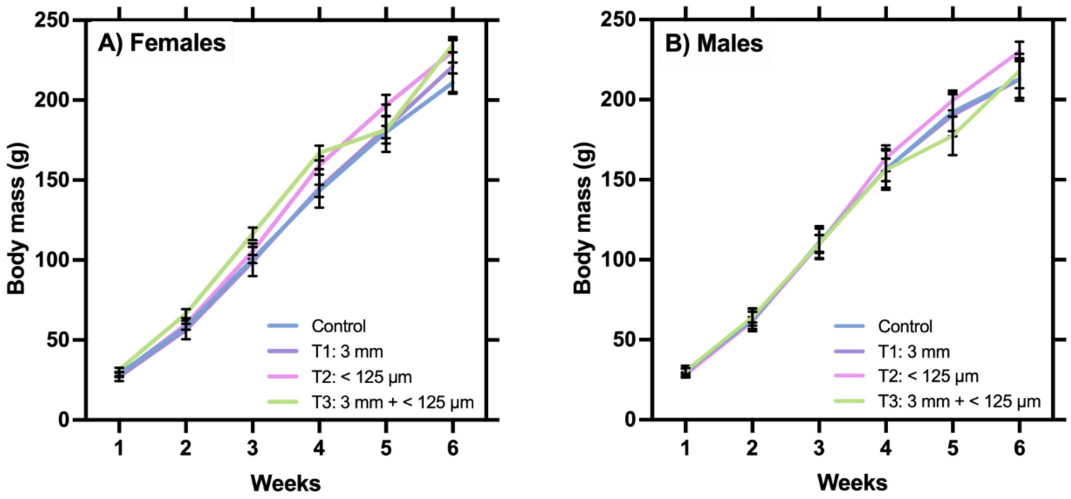

Fig. S15. *Body mass (g) of Japanese quail (Coturnix japonica) measured once per week in A) females (n = 32) and B) males (n = 23). The figure compares four treatment groups, Control (blue, n = 14), T1 (treatment 1: 3 mm particles, purple, n = 13), T2 (treatment 2: powder < 125 µm, pink, n = 14) and T3 (treatment 3: mixture of 3 mm and < 125 µm particles, green, n = 14). Error bars show standard error.*

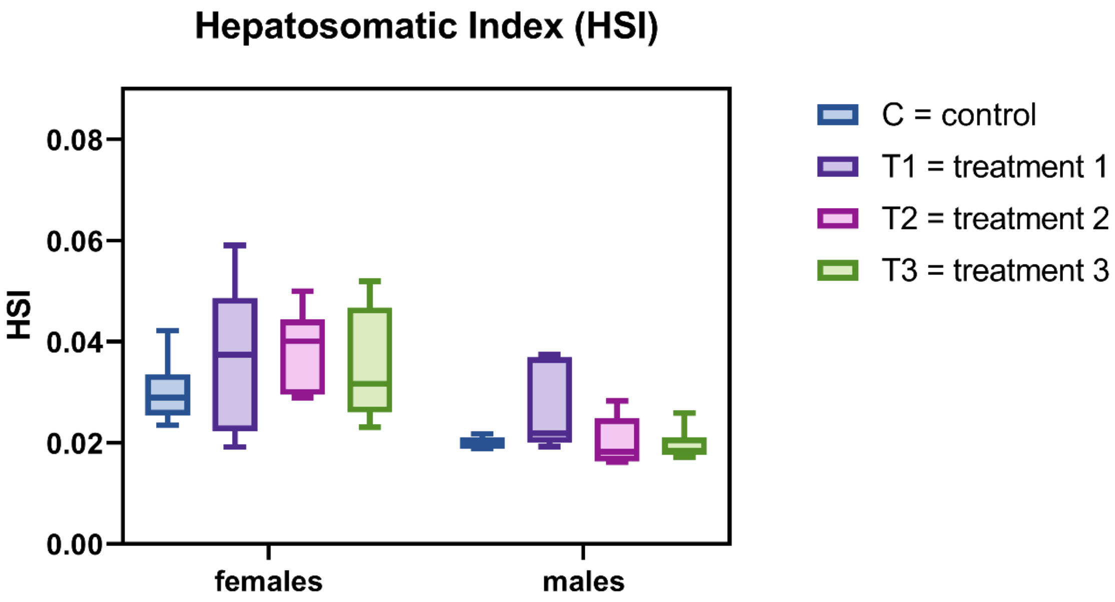

Fig. S16 *Boxplot showing Hepatosomatic Index (HSI) in Japanese quail (Coturnix japonica) for each treatment group (C n= 14, T1: 3 mm MP n=14, T2: < 125 µm MP n=13, T3: mixture 3 mm and < 125 µm MP n=14). As sex was an influential factor, the treatment groups are separated by sex.*

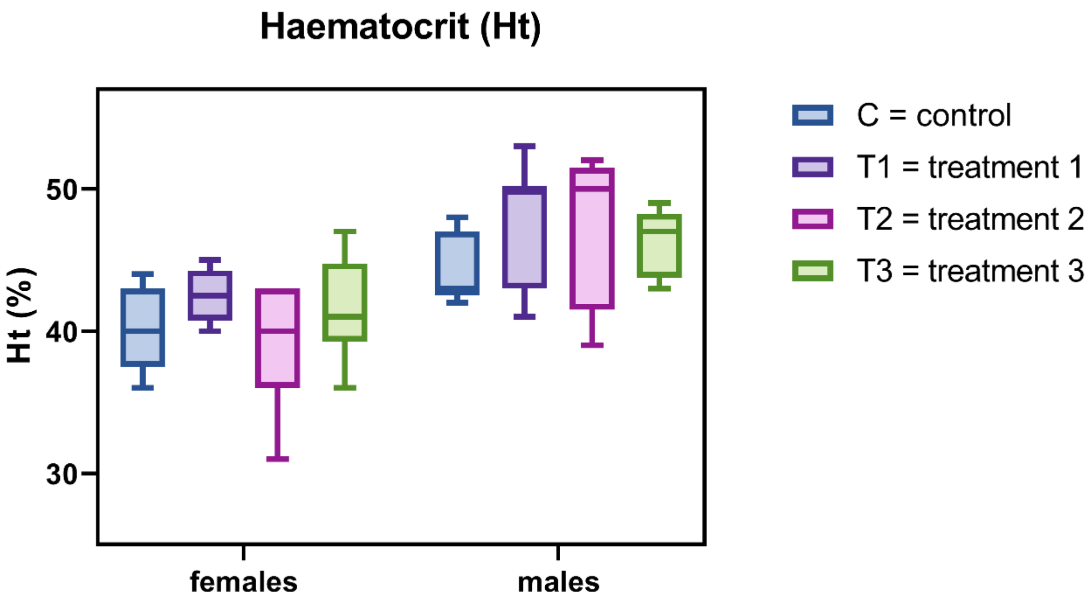

Fig. S17 *Boxplot showing Hematocrit (Ht, %) in Japanese quail (Coturnix japonica) for each treatment group (C n= 14, T1: 3 mm MP n=14, T2: < 125 µm MP n=13, T3: mixture 3 mm and < 125 µm MP n=14). As sex was an influential factor, the treatment groups are separated by sex.*

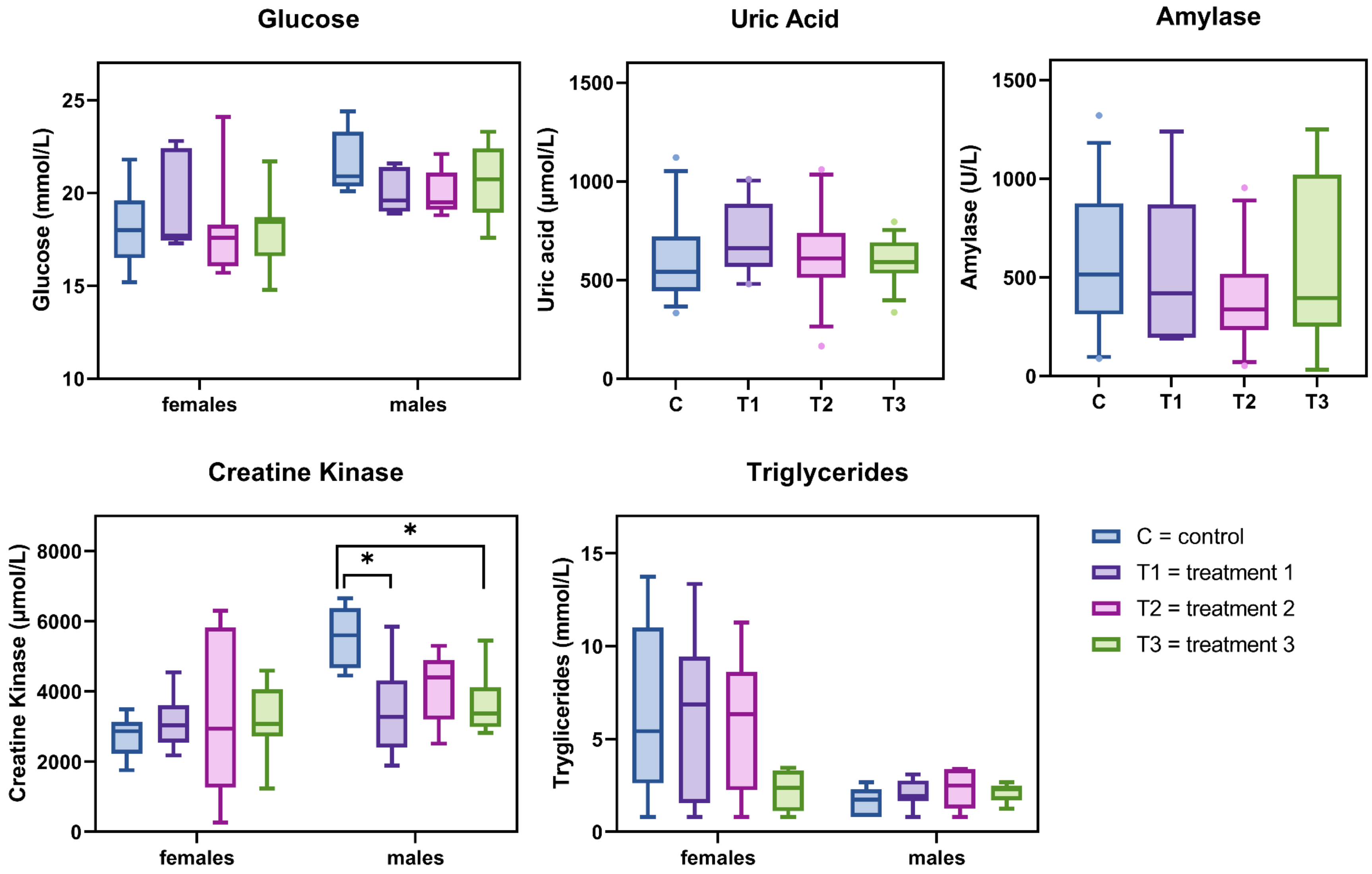

Fig. S18 *Boxplots showing BCCPs in blood of Japanese quail (Coturnix japonica) for each treatment group (C n= 14, T1: 3 mm MP n=14, T2: < 125 µm MP n=13, T3: mixture 3 mm and < 125 µm MP n=14). When sex was found to be an influential factor, the treatment groups are separated by sex. Statistical differences are shown (* P < 0.05).*

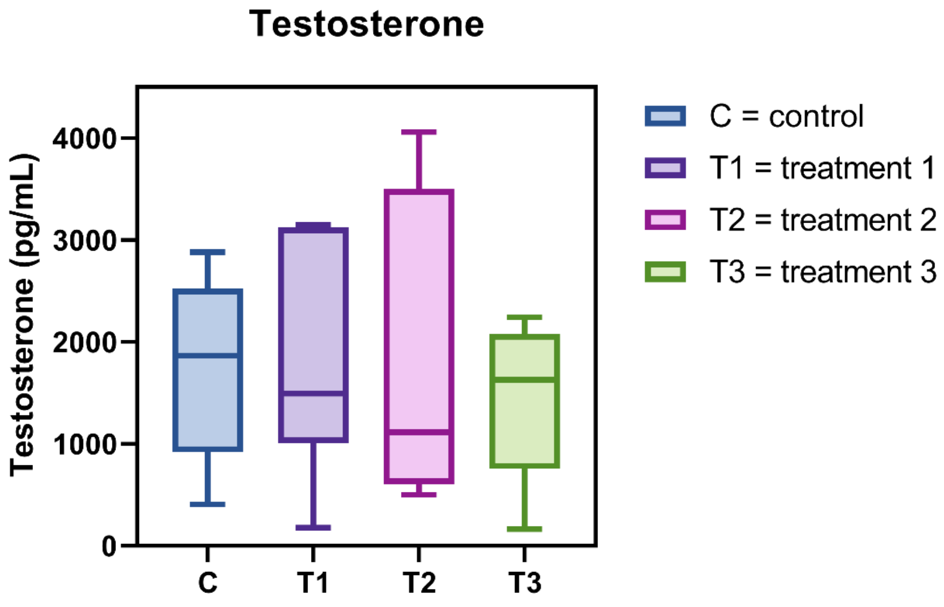

Fig. S19 *Boxplot showing testosterone levels in blood samples of male Japanese quail (Coturnix japonica) for each treatment group (C n= 14, T1: 3 mm MP n=14, T2: < 125 µm MP n=13, T3: mixture 3 mm and < 125 µm MP n=14).*

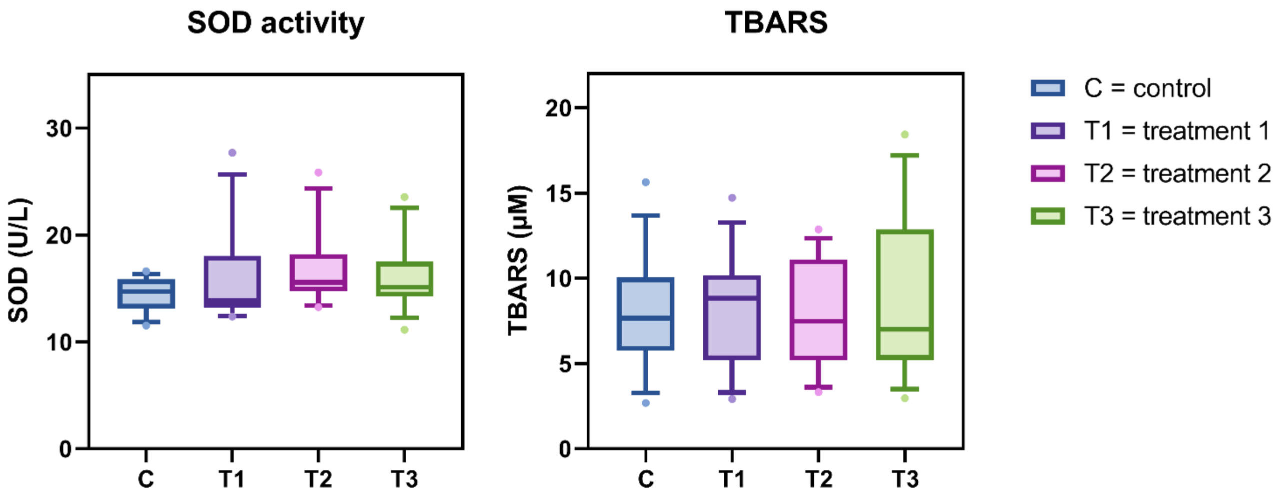

Fig. S20 *Boxplots showing oxidative stress parameters in blood of Japanese quail (Coturnix japonica) for each treatment group (C n= 14, T1: 3 mm MP n=14, T2: < 125 µm MP n=13, T3: mixture 3 mm and < 125 µm MP=14).*

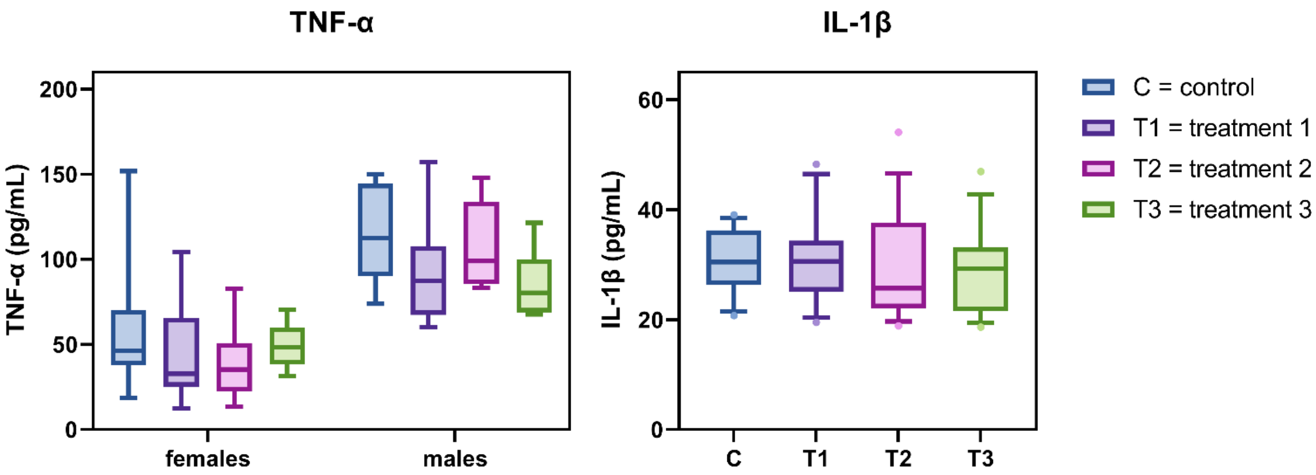

Fig. S21 *Boxplots showing cytokine levels in blood of Japanese quail (Coturnix japonica) for each treatment group (C n= 14, T1: 3 mm MP n=14, T2: < 125 µm MP n=13, T3: mixture 3 mm and < 125 µm MP n=14). When sex was found to be an influential factor, the treatment groups are separated by sex.*

References

(1) Huss, D.; Poynter, G.; Lansford, R. Japanese Quail (*Coturnix Japonica*) as a Laboratory Animal Model. *Lab Anim. (NY).* **2008**, *37* (11), 513–519. https://doi.org/10.1038/laban1108-513.

(2) Mennillo, E.; Cappelli, F.; Arukwe, A. Biotransformation and Oxidative Stress Responses in Rat Hepatic Cell-Line (H4IIE) Exposed to Organophosphate Esters (OPEs). *Toxicol. Appl. Pharmacol.* **2019**, *371* (April), 84–94. https://doi.org/10.1016/j.taap.2019.04.004.

(3) Supratman, S.; Purwanti, S.; Rahardja, D. P. Giving Bidara Leaf Extract (*Ziziphus Mauritiana*) through Drinking Water as an Alternative Antioxidant against Quail Haematological. *IOP Conf. Ser. Earth Environ. Sci.* **2021**, *788*. https://doi.org/10.1088/1755-1315/788/1/012066.
